# Nested Spatiotemporal Anomaly Detection with Semantic Augmentation: A Case Study in Heritage Conservation

**DOI:** 10.3390/s26185931

**Published:** 2026-09-19

**Authors:** Lidia Abad, Fernando Ramonet, Javier Ortega, José Javier Anaya, Sofía Aparicio

**Affiliations:** Institute of Physical and Information Technologies “Leonardo Torres Quevedo” (ITEFI), Consejo Superior de Investigaciones Científicas (CSIC), C/Serrano 144, 28006 Madrid, Spainsofia.aparicio@csic.es (S.A.)

**Keywords:** cultural heritage monitoring, wireless sensor networks, multivariate time series, anomaly detection, spatiotemporal deep learning, semantic anomaly detection

## Abstract

Cultural heritage (CH) sites are continuously exposed to pressures that can lead to deterioration. Continuous monitoring and anomaly detection (AD) enable early damage detection for preventive conservation. We propose a late-fusion AD framework combining NST-Net, a nested spatiotemporal neural network, with a semantic module encoding expert conservation rules, and evaluate it on five real-world CH monitoring datasets. NST-Net outperforms six state-of-the-art baselines on most sites, achieving, on average, 42% higher Average Precision than the best baseline per site under a synthetic evaluation protocol emphasizing sharp, short-duration anomalies. Dataset length serves as an important performance factor: the combined architecture and preprocessing pipeline particularly benefit longer campaigns, whereas they add little value on shorter ones. NST-Net also requires approximately 13 times less peak memory than baseline detectors, at a higher but still millisecond-scale latency. Further analysis shows that performance depends strongly on anomaly density, type, and severity. The semantic module passes label-free sanity checks and complements NST-Net effectively (complementarity index > 0.87), identifying conservation-relevant events missed by the deep model. These findings support late-fusion statistical–semantic frameworks for AD in CH monitoring.

## 1. Introduction

Cultural heritage (CH) assets embody irreplaceable evidence of human history and identity, and their degradation cannot be undone by reconstruction alone. Yet these assets are continuously exposed to environmental, structural, and anthropogenic stressors that accelerate deterioration and threaten long-term preservation. Climate variability, extreme environmental conditions, urban expansion, tourism, pollution, and insufficient maintenance can progressively compromise these heritage elements [1,2,3]. In many cases, degradation develops slowly and remains unnoticed until damage is already advanced, at which point remedial interventions are markedly more costly, more invasive, and less effective than preventive maintenance would have been. Continuous monitoring has therefore become a central component of preventive conservation, enabling the early detection of emerging risk conditions and supporting timely intervention [4].

Realizing this possibility in practice, however, is itself a data challenge. The growing deployment of Wireless Sensor Networks (WSNs) and digital monitoring infrastructures at heritage sites has produced large, heterogeneous data streams that exceed the capacity of manual inspection [5]. Automated analysis is therefore not merely convenient but essential for evidence-based conservation decision-making. Within this context, anomaly detection (AD) plays a central role in identifying deviations from expected behavior that may signal emerging conservation risks [6]. In heritage monitoring, however, meaningful deviations rarely occur in isolation: environmental, structural, and anthropogenic effects propagate across related sensors and develop over time. This makes the problem inherently multivariate and temporal, motivating the use of multivariate time series anomaly detection (MTSAD), where anomalies are identified by jointly modeling temporal patterns within individual sensors and dependencies across them [7].

Deep learning (DL) has substantially advanced the general MTSAD problem in recent years. Yet despite this progress, existing methods face three intertwined limitations when transplanted to CH monitoring. Most methods are developed and validated on benchmark datasets that are comparatively clean and stationary, whereas heritage sites exhibit pronounced non-stationarity and structured sensor unavailability [4]; standard evaluation protocols can substantially overestimate detector performance in the near-total absence of reliable ground truth [8]; and purely statistical detectors are, by construction, unable to flag conditions that are statistically normal yet operationally hazardous [9]. These three limitations, together with the corresponding prior work, are reviewed in Section 2.

Building on these limitations, this paper presents a framework for real-time MTSAD in CH monitoring under realistic operational conditions. Our contributions are fourfold:

1. We introduce NST-Net, a lightweight nested spatiotemporal architecture combining dilated temporal convolutions with multi-head feature attention to jointly learn reconstruction and forecasting tasks, incorporating an alive-mask mechanism that excludes inactive sensors from both the training objective and normalization statistics. NST-Net is benchmarked against six state-of-the-art baselines, and an ablation study identifies its most contributing elements (Section 4.1 and Section 4.2).
2. We design a rigorous evaluation protocol based on controlled synthetic anomaly injection and threshold-independent ranking metrics, providing a reproducible assessment of anomaly prioritization under label uncertainty. Both the injection protocol and metric selection are studied explicitly, including sensitivity to anomaly prevalence, type, and severity (Section 4.3).
3. We propose a late-fusion detection framework that complements the data-driven detector with a semantic reasoning module encoding site-specific expert conservation rules, enabling detection of operationally hazardous situations that remain statistically indistinguishable from normal behavior. We evaluate the stability and robustness of the semantic rules using label-free diagnostics and assess their complementarity with the DL detector (Section 4.4 and Section 4.5).
4. We evaluate the proposed framework on five real-world multivariate datasets collected within the ARGUS project [10], covering diverse environmental, structural, and anthropogenic monitoring conditions across five European heritage sites.

The remainder of this paper is organized as follows. Section 2 reviews related work on DL-based MTSAD and examines how existing approaches translate to CH monitoring, with particular attention to the challenges that arise in this setting. Section 3 describes the proposed framework, including the NST-Net architecture, the semantic-rule module, the datasets and preprocessing pipeline, and the evaluation methodology. Section 4 presents the experimental results: benchmarking against state-of-the-art baselines, an ablation study, evaluation-protocol sensitivity analysis, semantic-module diagnostics, and the late-fusion complementarity analysis. Section 5 discusses the limitations of the proposed approach, and Section 6 concludes this paper and indicates future lines of work.

## 2. Related Works

MTSAD methods have evolved considerably, yet their application to CH monitoring remains comparatively limited [11,12]. This section therefore reviews the broader MTSAD literature and evaluates the extent to which its methods address the distinctive requirements of long-term CH monitoring.

At a methodological level, MTSAD approaches differ first in how they define anomalous behavior. Reconstruction-based methods learn to reproduce normal observations and use reconstruction errors as anomaly scores, while forecasting-based methods learn temporal dynamics and identify deviations through prediction errors [13]. Hybrid approaches combine reconstruction and prediction objectives, whereas more recent methods derive anomaly scores from learned representations, association discrepancies, or deviations from expected temporal and cross-variable relationships [14,15,16]. These objectives are implemented through a range of temporal and spatiotemporal architectures. Recurrent models capture short-term sequential dependencies, while Temporal Convolutional Networks (TCNs) provide efficient modeling of local and multi-scale temporal patterns [17]. Transformer-based models extend this capability to longer-range temporal dependencies, and graph- and attention-based methods explicitly model relationships between variables, combining temporal dynamics with cross-sensor dependencies [16,18,19,20]. Thus, contemporary MTSAD research spans different detection objectives and architectural choices rather than following a single modeling paradigm.

For CH monitoring, however, the relevance of these approaches depends not only on their detection objective or architecture but also on the conditions under which they are trained and deployed. Most MTSAD methods are developed and evaluated on benchmark datasets that are comparatively clean, stationary, and fully observed [8,16]. In contrast, long-term heritage monitoring is characterized by seasonal cycles, long-term trends, sensor drift, and heterogeneous acquisition conditions, resulting in temporal distribution shifts and structured sensor unavailability. Intermittent sensor failures, for example, can produce non-random missingness, while sensor configurations and environmental conditions may vary substantially across sites [21,22]. These conditions can alter both the temporal patterns and cross-sensor relationships learned during training, making robustness to non-stationarity and partial observations an important requirement for applying MTSAD to CH monitoring.

A further challenge concerns how MTSAD methods are evaluated. Common Point-Adjusted (PA) metrics can overestimate detection performance by assigning full credit to an anomalous segment when only a small portion of it is correctly detected [8,23]. More fundamentally, benchmark datasets generally provide ground-truth anomaly labels, whereas such labels are rarely available for long-term CH monitoring. Conservation hazards may be latent, inconsistently documented, or only recognized after visible deterioration has occurred. This makes conventional supervised evaluation difficult to reproduce in practice and motivates evaluation strategies that explicitly account for label scarcity, including controlled synthetic anomaly injection and threshold-independent ranking measures.

Beyond distributional deviations, CH monitoring also requires identifying conditions that may remain statistically consistent with previously observed behavior but nevertheless represent a conservation risk. Statistical anomaly detectors are inherently limited in this respect because their scores are derived from deviations from learned data distributions [24,25,26]. In heritage applications, however, the significance of an observation can depend on its physical context, conservation requirements, or combinations of environmental and structural conditions [27]. Knowledge-augmented approaches therefore provide a complementary means of detecting semantically meaningful risks by incorporating explicit domain constraints or expert rules [9]. Comparable interpretable, rule-augmented DL frameworks have also been proposed in other safety-critical monitoring domains, reinforcing the broader relevance of combining data-driven detectors with domain-informed structure beyond the CH monitoring context [28].

## 3. Materials and Methods

For clarity, all mathematical notation introduced throughout this section is summarized in Table A1 (Appendix A).

### 3.1. Problem and System Overview

Let $X\in R^{N\times F}$ denote a complete multivariate time series with *N* timestamps and *F* variables. Given a lookback window of length *T*, the input associated with time *t* is defined as $x_{t}=X_{t-T+1:t}\in R^{T\times F}$. The goal of MTSAD is to assign to each window $x_{t}$ a continuous anomaly score $s_{t}\geq 0$ and a binary label $y_{t}\in \left\{0,1\right\}$, where $y_{t}=1$ indicates an anomalous window. The anomaly score is continuous and thresholded to obtain the binary label.

**Figure 1:**
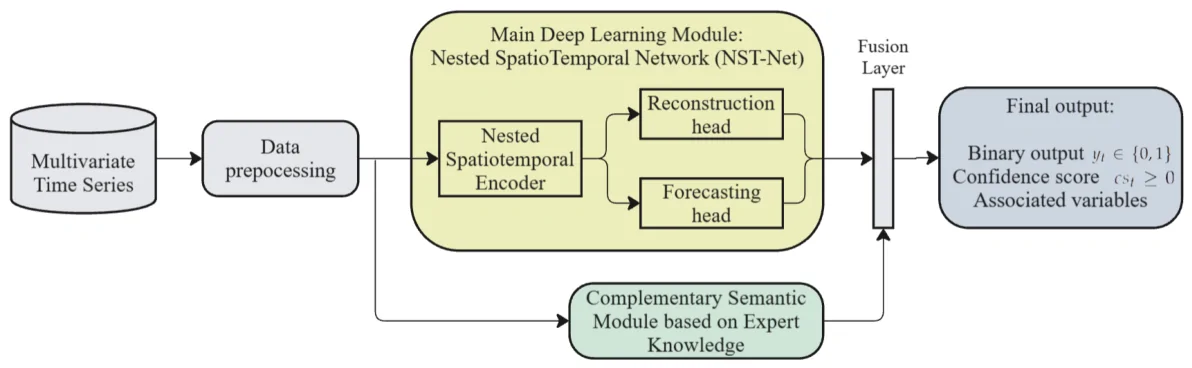
Pipeline of the system.

The primary detector is NST-Net, which jointly models temporal dynamics and inter-variable dependencies through a nested spatiotemporal architecture. The network is trained using a joint reconstruction–forecasting objective, allowing complementary anomaly evidence to be extracted from both tasks. A detailed description of the architecture is provided in Section 3.2. A fundamental limitation of purely data-driven models is their inability to detect hazardous states that lie within the learned distribution. Many conservation-relevant risks are statistically normal yet operationally dangerous [25,26]. To address this, a complementary semantic module encodes expert-defined rules targeting these in-distribution risks. The outputs of both modules are fused into a unified detection signal. The final system output comprises (i) a binary label $y_{t}\in \left\{0,1\right\}$; (ii) a confidence score $cs_{t}\geq 0$, defined only when $y_{t}=1$ and expressed as $cs_{t}=f\left(s_{t}\right)$; and (iii) the set of contributing variables and their relative contribution, reported only when $y_{t}=1$. Figure 1 illustrates the overall pipeline. The dataset is preprocessed and fed to both the main DL module and the complementary semantic module. Their outputs are then fused to generate the final output. Practical deployment guidelines are provided in Appendix B. The complete framework, including NST-Net, the semantic-rule module, and the evaluation pipeline, was implemented in Python (v3.12.3) using PyTorch (v2.5.1+cu118); figures were generated using Matplotlib (v3.10.0).

### 3.2. Deep Learning Model

#### 3.2.1. Architecture

**Figure 2:**
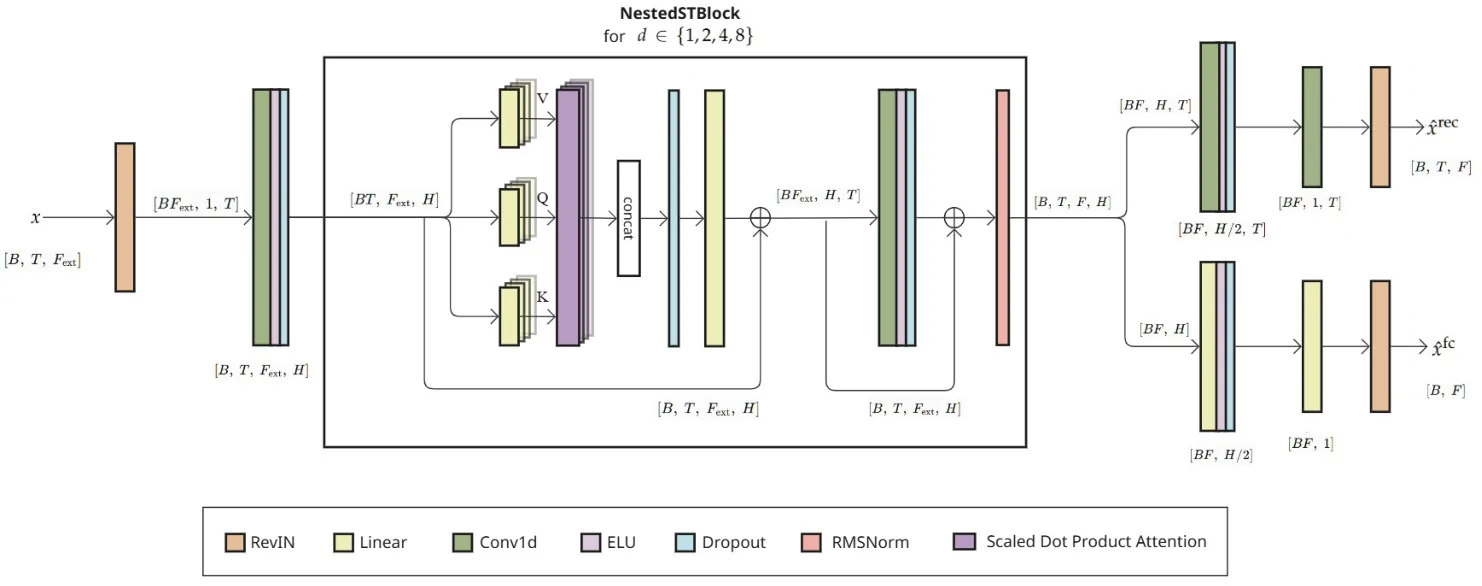
NST-Net architecture. *x* denotes the input window, ${\hat{x}}^{\mathrm{rec}}$ denotes the reconstructed window, and ${\hat{x}}^{\mathrm{fc}}$ denotes the forecasted output. V, Q, and K refer to the values, queries, and keys of the attention mechanism. The NestedSTBlock is applied four times, once for each dilation factor d∈{1,2,4,8}. Tensor shapes include *B* as batch, *F* as features, $F_{ext}$ as the extended feature set, *T* as the lookback window length, and *H* as the hidden dimension.

The anomaly detector is based on the proposed NST-Net, which jointly models temporal dynamics and cross-variable dependencies. Following the taxonomy in [16], NST-Net belongs to the nested temporal–spatial dependency category, where temporal and spatial relationships are learned iteratively rather than through independent parallel branches or fixed sequential pipelines. Specifically, the encoder comprises four nested spatiotemporal stages, each combining a multi-head feature self-attention module with a dilated TCN. At every stage, the spatial representation is first refined through self-attention to capture inter-variable dependencies and subsequently processed by dilated temporal convolutions to learn multi-scale temporal patterns. Repeating this spatial-to-temporal refinement enables both types of dependencies to co-evolve throughout the network, progressively producing increasingly informative latent representations. The final representation feeds two complementary output branches: (i) a reconstruction branch that reconstructs the input window and (ii) a one-step forecasting branch that predicts the subsequent observation. Combining both objectives allows the model to detect complementary forms of anomalous behavior: reconstruction errors are more sensitive to persistent structural deviations, whereas forecasting errors better capture abrupt changes in system dynamics, yielding a more robust anomaly score than either objective alone [16]. Figure 2 illustrates the complete NST-Net architecture.

The input is defined as $x\in R^{B\times T\times F_{\mathrm{ext}}}$, where *B* denotes the batch size, *T* the lookback window length, and $F_{\mathrm{ext}}=F+F_{c}$ the total number of input variables, with *F* denoting the sensor variables and $F_{c}=6$ the temporal context features (sine and cosine encodings of hour-of-day, day-of-week, and day-of-year, described in Section 3.4.3). Reversible Instance Normalization (RevIN) [29] is applied exclusively to the *F* sensor variables; the $F_{c}$ context features are passed through unchanged, as their range is fixed by construction and instance normalization of periodic encodings is not meaningful. Unlike the original formulation, the normalization center is computed using the temporal median rather than the mean, improving robustness to anomalous observations, as a single anomaly inside a window can pull the mean but not the median. The normalized input is projected into a latent representation of dimension H=32 through a pointwise convolution (k=1, Conv1d), followed by Exponential Linear Unit (ELU) activation [30] and dropout with rate 0.3.

The resulting representation is processed by a nested spatiotemporal encoder composed of successive dilation stages with dilation factors d∈{1,2,4,8}. At each stage, multi-head self-attention with four heads is applied across variables at every time step, enabling the model to capture dynamic inter-variable dependencies. The attention mechanism operates on feature embeddings. Representations are linearly projected into queries (Q), keys (K), and values (V) via learned transformations. Scaled dot-product attention is applied across variables, and the resulting attention weights are used to compute a weighted aggregation of value vectors. Dropout is applied to the attention outputs for regularization, and a final linear projection maps the aggregated representations back to the latent space. The output is integrated with the input via a residual connection.

Subsequently, a causal dilated temporal convolution with kernel size k=5 is applied independently to each variable. The choice of k=5 ensures that the receptive field spans the full periodic window of one day without increasing model depth, given that the encoder already includes four dilation stages. Causality is enforced via asymmetric left padding of size (k−1)d, ensuring that each representation depends only on current and past observations. The convolution output is passed through ELU activation and dropout and is again added via a residual connection, followed by Root Mean Square Layer Normalization (RMSNorm) [31]. RMSNorm preserves the low-frequency structure by avoiding mean subtraction, preventing the trend-loss effect introduced by LayerNorm.

The encoder output is passed to two complementary output branches operating only on the *F* target variables. The $F_{c}$ context feature representations contribute only through the encoder’s attention mechanism and are discarded at the output stage. The reconstruction branch reconstructs the full input window ${\hat{x}}^{\mathrm{rec}}\in R^{B\times T\times F}$ using a lightweight two-layer pointwise convolutional decoder with bottleneck dimension H/2, ELU activation, and dropout. The forecasting branch predicts the next time step ${\hat{x}}^{\mathrm{fc}}\in R^{B\times F}$ using a two-layer linear decoder with the same bottleneck structure. Both outputs are denormalized using the inverse RevIN transformation before loss computation.

All weights are initialized using Xavier initialization [32]. Training is performed for 250 epochs using the Adam optimizer [33] with learning rate $10^{-3}$ and weight decay $10^{-4}$. A learning-rate scheduler reduces the learning rate by a factor of 0.5 after 5 epochs without improvement on the validation loss. Early stopping with a patience of 20 epochs is applied to prevent overfitting. Hyperparameters were selected via preliminary experiments on a held-out validation set.

#### 3.2.2. Training and Validation

The model is trained with a weighted multi-task loss, commonly used in the literature [16], defined as

(1) $$L=\beta \;L_{\mathrm{rec}}+\left(1-\beta \right)\;L_{\mathrm{fc}},$$

where β∈[0,1] controls the trade-off between the two tasks. Each term is a trimmed masked L1 loss. L1 is preferred over L2 loss because the combination of reconstruction-based modeling and instance normalization (RevIN) produces residuals of small magnitude during training, causing L2-based objectives to yield vanishing gradients. Let $B_{\mathrm{rec}}$ and $B_{\mathrm{fc}}$ denote the sets of the ⌈(1−ρ)B⌉ windows with the lowest per-window losses for the reconstruction and forecasting terms, respectively. The trimmed batch losses are

(2) $$L_{\mathrm{rec}}=\frac{1}{|B_{\mathrm{rec}}|}\sum_{b\in B_{\mathrm{rec}}}\ell_{b}^{\mathrm{rec}},\;L_{\mathrm{fc}}=\frac{1}{|B_{\mathrm{fc}}|}\sum_{b\in B_{\mathrm{fc}}}\ell_{b}^{\mathrm{fc}}.$$

where ρ=0.05 is sufficiently large to reduce the risk of the model learning to reconstruct or forecast anomalous behavior. Let $M\in {\left\{0,1\right\}}^{B\times T\times F}$ denote the alive mask (explained in Section 3.4.2), where M=1 indicates a valid observation. The authors define the per-window reconstruction loss as an aggregate of errors over the full window, while the forecasting loss is evaluated at the single target timestep immediately following the end of the input window:

(3) $$\ell_{b}^{\mathrm{rec}}=\frac{\sum_{t=1}^{T}\sum_{f=1}^{F}M_{b,t,f}\;\left|x_{b,t,f}-{\hat{x}}_{b,t,f}^{\mathrm{rec}}\right|}{\sum_{t=1}^{T}\sum_{f=1}^{F}M_{b,t,f}+\varepsilon },\;\ell_{b}^{\mathrm{fc}}=\frac{\sum_{f=1}^{F}M_{b,f}\;\left|x_{b,T+1,f}-{\hat{x}}_{b,T+1,f}^{\mathrm{fc}}\right|}{\sum_{f=1}^{F}M_{b,T+1,f}+\varepsilon }$$

#### 3.2.3. Inference

At inference time (with B=1), the DL anomaly score is defined, similarly to Equation (1), as a weighted combination of a reconstruction and a forecasting term:

(4) $$s^{\mathrm{DL}}=\beta \;s^{\mathrm{rec}}+\left(1-\beta \right)\;s^{\mathrm{fc}},$$

where β is the same as in Equation (1). The authors derive the reconstruction score by aggregating normalized reconstruction residuals over the lookback window using an Exponential Moving Average (EMA, α=0.5) [34] and retaining its final state to emphasize recent observations and reduce detection latency. The forecasting score is derived as the average normalized one-step prediction error across all features:

(5) $$s^{\mathrm{rec}}=\frac{1}{F}\sum_{f=1}^{F}\underset{t}{\mathrm{EMA}}\;\left(z_{t,f}^{\mathrm{rec}}\right),\;s^{\mathrm{fc}}=\frac{1}{F}\sum_{f=1}^{F}z_{f}^{\mathrm{fc}},$$

with normalized residuals defined by the authors as

(6) $$z_{t,f}^{\left(\cdot \right)}=\frac{e_{t,f}^{\left(\cdot \right)}}{n_{f}+\varepsilon },\;\left(\cdot \right)\in \left\{\mathrm{rec},\mathrm{fc}\right\},$$

where $e_{t,f}^{\mathrm{rec}}$ and $e_{t,f}^{\mathrm{fc}}$ are the reconstruction and forecasting errors, respectively. The feature scale $n_{f}$ is defined as the difference between the 99th and 1st percentiles of feature *f*, computed over the combined training and validation sets. This robust normalization avoids assuming a common parametric distribution, since the monitored variables exhibit markedly different statistical behaviors.

A threshold is required to construct the binary output of the module (Section 3.1). This threshold τ is estimated from the training and validation score distributions using a robust median-based estimator:

(7) $$\tau =\mathrm{median}\left(s^{\mathrm{DL}}\right)+3\times 1.4826\times \mathrm{MAD}\left(s^{\mathrm{DL}}\right),$$

where MAD denotes the median absolute deviation [35]. The factor 1.4826 converts the MAD into a robust estimate of the standard deviation under Gaussian assumptions, and the multiplier 3 corresponds to the conventional three-sigma threshold used for AD. The binary detection output is

(8) $$y_{t}^{\mathrm{DL}}=\left\{\begin{array}{cc} 1, & s_{t}^{\mathrm{DL}}\geq \tau ,\\ 0, & \mathrm{otherwise}, \end{array}\right.$$

where $s_{t}$ corresponds to the model output for the window ending at time *t*. The authors derive a confidence score for each detected DL event as

(9) $$cs^{\mathrm{DL}}=0.5+0.5\left(1-e^{-\frac{s^{\mathrm{DL}}-\tau }{s_{\mathrm{tail}}}}\right),$$

where $s_{\mathrm{tail}}$ is the standard deviation of the training and validation scores exceeding the threshold. Consequently, $cs^{\mathrm{DL}}\in \left[0.5,1\right)$ for $y^{\mathrm{DL}}=1$, with larger values indicating more confident detections.

### 3.3. Complementary Semantic Module

The semantic module complements the DL detector by identifying hazardous conditions that may remain statistically normal but are known to be relevant for CH conservation. It consists of a set of expert-defined rules ${\left\{r_{j}\right\}}_{j=1}^{J}$, each associated with a subset of monitored features and derived in collaboration with domain experts for each pilot site (Appendix C).

For each rule $r_{j}$, a rule-specific function $g_{j}\left(\cdot \right)$ computes an activation value $z_{t,j}=g_{j}\left(x_{t}\right)$, where $g_{j}\left(\cdot \right)$ depends on the rule semantics (e.g., persistence above a threshold, cumulative exceedance, or temporal variability). The activation is then converted into a rule score $s_{t,j}\in \left[0,1\right]$ using one of two scoring schemes proposed by the authors. For rules with expert-annotated hazard intervals, thresholds are estimated directly from the observations within the annotated period. Let $Q_{0.01}$ and $Q_{0.99}$ denote the empirical 1st and 99th percentiles of the corresponding feature computed over the labeled interval. The rule score is defined as

(10) $$s_{t,j}=\left\{\begin{array}{cc} 1, & z_{t,j}<Q_{0.01}\;\mathrm{or}\;z_{t,j}>Q_{0.99},\\ 0, & \mathrm{otherwise}, \end{array}\right.$$

where the percentile thresholds provide robust, data-driven bounds without requiring assumptions on the underlying distribution. Note that this strategy is used because only the event date is known, rather than the specific triggering feature or exact timing. For rules without annotated hazard intervals, the activation is mapped to a continuous confidence score through

(11) $$s_{t,j}=\sigma \;\left(k_{j}\left(z_{t,j}-\theta_{j}\right)\right),$$

where $\theta_{j}$ is the expert-defined activation threshold for rule $r_{j}$ (Appendix C), and $k_{j}$ is a rule-specific scaling factor, defined as the inverse of the corresponding feature standard deviation, to obtain comparable sensitivities across rules. Table 1 presents the semantic-rule set of one of the pilot sites of the ARGUS project (P1) together with the corresponding scoring scheme. The complete rule definitions for all pilot sites are provided in Appendix C.

The final semantic anomaly score is defined by the authors as

(12) $$s_{t}^{\mathrm{SEM}}=\max_{j}s_{t,j},$$

corresponding to a logical OR over all rule scores. In the semantic module, a window is defined as anomalous whenever

(13) $$y_{t}^{\mathrm{SEM}}=\left\{\begin{array}{cc} 1, & s_{t}^{\mathrm{SEM}}\geq 0.5,\\ 0, & \mathrm{otherwise}. \end{array}\right.$$

As the continuous anomaly score is already normalized and uses a common threshold of 0.5, the confidence score is defined as $cs_{t}^{\mathrm{SEM}}=s_{t}^{\mathrm{SEM}}$ without further scaling. The contributing variables are those involved in rule activation at time *t*.

### 3.4. Datasets and Preprocessing

#### 3.4.1. Study Sites and Data Acquisition

This study introduces five operational monitoring datasets collected within the ARGUS project across heterogeneous CH sites in Europe: Delos Island (P1, Greece), Baltanás Cellar Town (P2, Spain), Monti Lucretili Natural Park (P3, Italy), Sant’Antonio di Ranverso Abbey (P4, Italy), and Schenkenberg Castle (P5, Switzerland). The datasets were acquired under real deployment conditions between September 2025 and June 2026, covering monitoring periods ranging from 2 to 10 months and comprising more than 75 sensors.

Each site integrates three sensing modalities. Environmental variables include temperature, relative humidity, precipitation, wind direction and speed, light intensity, ultraviolet radiation, barometric pressure, gas concentration, and particulate matter. Structural variables include vibration, inclination, displacement, crack evolution, and moisture. Anthropogenic variables include people and vehicle counters. This multimodal structure introduces complex cross-domain dependencies that motivate the joint spatiotemporal treatment adopted in Section 3.2. Acquisition frequencies vary substantially both across and within sites, from event-based counters to periodic measurements ranging from 1 min to 1 day, with more than 30 raw variables recorded per site. This heterogeneity in modality, sampling rate, and sensor type is characteristic of real-world WSN deployments and is not typically represented in standard MTSAD benchmarks, motivating the preprocessing and modeling choices described in the remainder of this section.

#### 3.4.2. Shared Preprocessing Pipeline

All datasets undergo the same preprocessing pipeline before being used by any component of the proposed framework. Sensor data originating from multiple CSV files and acquisition formats are first loaded and merged into a unified multivariate time series. Variable names and timestamps are standardized through namespace harmonization, duplicate removal, flexible datetime parsing, and automatic timestamp normalization. Measurements belonging to the same physical sensor are subsequently merged while preserving temporal consistency.

A first cleaning stage (Figure 1) removes redundant cumulative variables whenever their non-cumulative counterparts are available, preventing duplicated measurements with different noise characteristics. Variables with an alive ratio below 10% are discarded due to insufficient temporal coverage. Angular measurements are converted into sine and cosine components to preserve periodic continuity, while sensors affected by relocation events are split into independent pre- and post-relocation variables to account for changes in their physical measurement context. Site-specific deterministic corrections are then applied to remove known acquisition artifacts before statistical processing. Depending on the deployment, these include removing corrupted intervals, filtering physically implausible values, rescaling abnormal measurement ranges, and converting cumulative measurements into incremental quantities. These corrections ensure that known acquisition issues are explicitly handled rather than being interpreted as anomalies.

A frozen-sensor detection stage is subsequently applied to the original temporal signals before resampling. Physical sensors are first identified by grouping their associated variable columns under a common sensor identifier; a sensor is automatically flagged as inactive whenever all variables belonging to that identifier are simultaneously missing for a contiguous period of at least three days, a threshold set above the longest expected native reporting interval across the deployed sensor types, so that a single missed transmission cycle does not trigger a false freeze flag. Once a qualifying gap is detected, the corresponding frozen window is conservatively extended by one additional day before the sensor is allowed to be marked alive again. Automatically detected intervals are complemented with manually curated freeze periods identified by domain experts for each deployment (e.g., communication failures, battery depletion, vandalism, and sensor trials excluded from analysis), encoded as explicit per-sensor date ranges and merged with the automatic detection into a single alive mask. A dedicated rule additionally flags implausible thermistor readings above a fixed magnitude threshold as invalid, treating a contiguous run of invalid readings as a single episode rather than fragmenting it into many short gaps. This was done after several thermistors suffered shifts in their readings. Finally, for variables with an explicitly registered valid window (e.g., before/after a sensor relocation or a manually delimited sub-range), missing values outside that window are folded into the alive mask as well, preventing such variables from being read as available when they are, by design, out of scope. Frozen intervals are replaced with missing values, and an alive mask *M* is generated to indicate sensor availability over time. This mask is subsequently used throughout the framework to exclude inactive sensors during expert-rule evaluation, feature normalization, DL loss computation, and performance evaluation. The alive ratio reported in Table 2 corresponds to the mean value of *M* across time and variables. Although effective at identifying complete sensor failures, the current implementation does not detect gradual sensor degradation that still produces varying measurements.

Finally, the datasets are resampled to a common 30 min grid to provide a unified temporal representation across sites with heterogeneous acquisition frequencies. Continuous variables are averaged within each 30 min interval, while counter-type variables are aggregated by summation. Missing values in the resampled continuous signals are subsequently time-interpolated for a maximum of 48 consecutive 30 min samples (24 h). This tolerance was selected to accommodate a specific class of low-frequency sensors used at the P2 site. These temperature and humidity sensors, installed inside the Baltanás cellars, record approximately once per day or when the measured variable changes beyond a predefined percentage. Their low acquisition frequency reflects the highly stable environmental conditions of the cellars, which were designed to maintain relatively constant temperature and humidity. As shown in Table 2, P2 is the only pilot site containing sensors with an acquisition frequency of approximately one day. The 24 h interpolation tolerance therefore allows these legitimate low-frequency measurements to be represented on the common 30 min grid. It should also be noted that the other monitored variables have substantially higher acquisition frequencies; therefore, the 24 h interpolation tolerance is not intended to bridge their normal acquisition intervals. Their availability is additionally tracked through the sensor alive mask, which is used downstream to distinguish valid observations from periods of sensor unavailability. Nevertheless, interpolation cannot recover physical changes that develop entirely within an unobserved interval and may therefore attenuate slow cumulative processes, such as moisture drift or gradual structural movement. The resampling procedure is consequently intended to harmonize heterogeneous acquisition frequencies rather than reconstruct long-term physical degradation processes.

#### 3.4.3. DL Model-Specific Input Preparation

After the shared preprocessing stage, additional transformations are applied specifically for the DL model. First, six temporal context variables ($F_{c}=6$) are appended to the input, corresponding to sine and cosine encodings of hour-of-day, day-of-week, and day-of-year. Environmental monitoring data exhibit strong periodic behavior driven by known temporal cycles that cannot be inferred from sensor measurements alone. Providing these variables explicitly allows the network to condition its representation of normal behavior on the current temporal context. Sine–cosine encoding preserves cyclic continuity at period boundaries [29]. Since these variables are already bounded within [−1,1], they are excluded from subsequent normalization.

The data are then partitioned chronologically into training, validation, and test sets using a fixed ratio of 0.8:0.1:0.1, preventing temporal leakage. Variables whose alive ratio falls below 10% in any split are discarded to ensure reliable statistics during optimization. The resulting number of variables for each pilot site is reported in Table 2. Input samples are finally generated using overlapping sliding windows of length T=72 (36 h) and stride s=1, covering more than one complete daily cycle.

To address both sensor heterogeneity and temporal non-stationarity, a two-stage normalization strategy is adopted. Global feature normalization is first performed using a StandardScaler after clipping observations to their 1st and 99th percentiles, consistent with the anomaly-score formulation described in Section 3.2.3. Feature statistics are computed exclusively from valid observations identified by the alive mask *M*. Standardization was preferred over MinMax scaling, which proved highly sensitive to outliers, and RobustScaler, whose median- and interquartile-based statistics were less informative for the highly skewed and zero-inflated distributions commonly observed in the monitored variables. To mitigate the strong temporal non-stationarity exhibited by the monitored heritage environments, RevIN [29] is incorporated as an online normalization mechanism. The architectural details of the proposed RevIN implementation are presented in Section 3.2.1.

#### 3.4.4. Dataset Summary Statistics

Table 2 summarizes dataset-level statistics before and after the processing stages introduced in Section 3.4.2 and Section 3.4.3. The number of variables is denoted as #Vars (Raw) for the original set of variables, #Vars (Clean) for the variables retained after initial preprocessing (Section 3.4.2), and #Vars (DL-Ready) for the variables used by the DL module (Section 3.4.3). The sets of Clean and DL-Ready variables are classified into environmental (Env.), structural (Str.), and anthropogenic (Ant.) variables. The duration of the data acquisition campaigns, along with the range and predominant sampling period, is reported. The number of samples is shown before and after resampling to a common 30 min frequency through #Samples Raw and #Samples Clean, respectively. The alive ratio denotes the proportion of valid sensor–timestep observations computed over the clean variable set.

**Figure 3:**
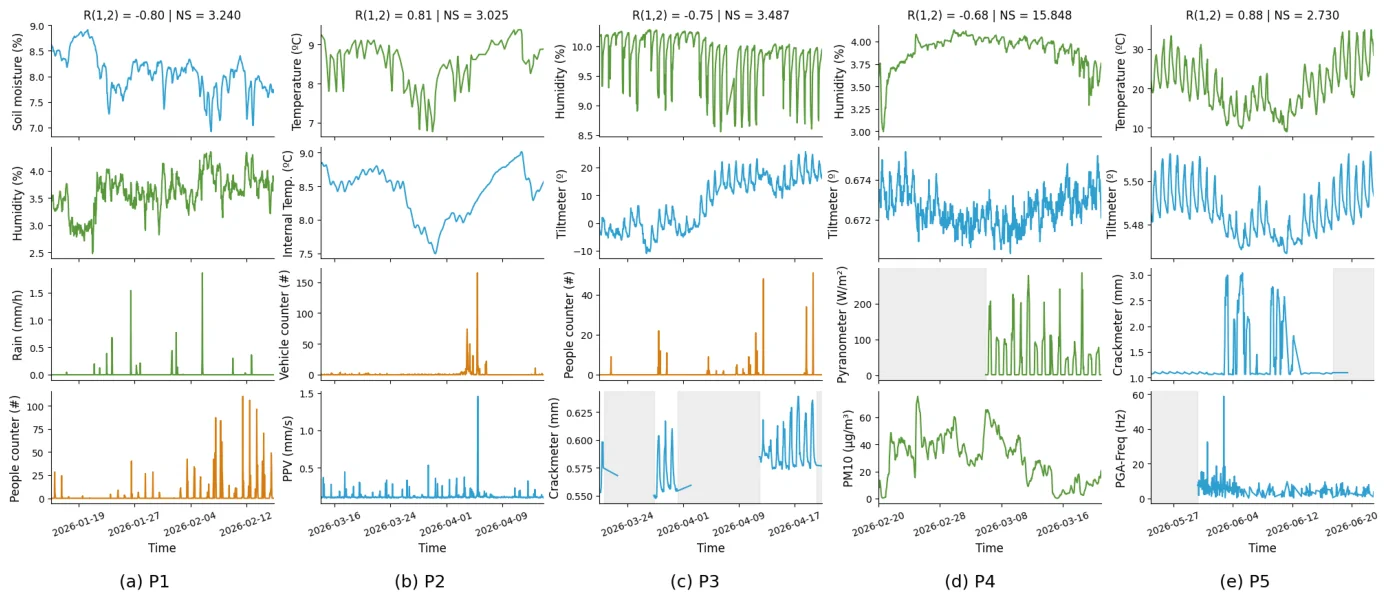
Representative time series of four variables across the five datasets (P1–P5). Colors indicate environmental (green), structural (blue), and anthropogenic (orange) variables; gray marks missing data. R(1,2) is the Spearman correlation between the first two features, and NS is the non-stationarity index of the four features shown for each dataset.

Figure 3 shows, for each site, four representative time series selected from the full variable set due to space constraints, color-coded by modality: environmental (green), structural (blue), and anthropogenic (orange). Three characteristics stand out. First, all sites exhibit strong temporal non-stationarity: environmental variables show regime shifts and seasonal drift, structural variables display slow trends punctuated by abrupt changes, and anthropogenic signals alternate between quiescent and bursty regimes. The non-stationarity index (NS), defined in this work to quantify the temporal variability of the four variables shown, ranges from 2.730 (P5) to 15.848 (P4). Second, extended missing segments (gray shading) reflect sensor outages and communication failures, consistent with the alive ratios reported in Table 2, which remain below 0.75 at every site. Third, pronounced cross-modal dependencies are observed, with Spearman correlations [36] between selected variable pairs ranging from −0.80 to 0.88, underscoring the need to model inter-variable structure rather than treating sensors independently. Together with Table 2, these observations motivate the two core modeling requirements addressed in Section 3.2: robustness to structured, high-rate missingness and joint spatiotemporal representation of heterogeneous, non-stationary signals.

### 3.5. Evaluation

The evaluation is organized into five sections. First, NST-Net is benchmarked against representative state-of-the-art MTSAD methods (Section 4.1). Baseline comparisons include methods spanning distinct modeling paradigms: Isolation Forest [37], a non-temporal tree-based detector; USAD [38], an adversarially trained autoencoder; GDN [39], a graph-based detector relying on a learned static sensor graph; MTAD-GAT [18], which combines temporal and feature-wise graph attention; Anomaly Transformer [14], which exploits association-discrepancy modeling; and TimesNet [40], which captures multi-periodicity through 2D temporal variation. This selection covers the main architectural families used in current MTSAD research and commonly used baselines, allowing NST-Net to be benchmarked against both classical and state-of-the-art approaches. To ensure a fair comparison, all baseline models were trained using the same early-stopping criterion as NST-Net (patience of 20 epochs on validation loss, with a maximum of 250 epochs) and with hyperparameters selected using the common tuning protocol described in Appendix D. However, restrictions in some baseline implementations prevented the inclusion of several components of the NST-Net preprocessing pipeline, including RevIN, cyclic temporal features $F_{c}$, and the alive mask *M*. To disentangle the two sources of NST-Net’s advantage, the architecture itself versus its preprocessing pipeline, a matched baseline-preprocessing variant of NST-Net (NST-Net-bp) is evaluated, in which RevIN, the temporal context features, and the alive mask are removed and missing values are imputed identically to the preprocessing pipeline of the baselines. In addition, to assess suitability for real-time deployment, computational efficiency (trainable parameters, per-window inference latency, and peak memory) is reported for NST-Net and all baselines. All measurements were obtained on the same workstation, equipped with an NVIDIA GeForce RTX 4060 Ti (16 GB VRAM; NVIDIA Corporation, Santa Clara, CA, USA), using the same software environment and inference configuration for all models. Second, an ablation study quantifies the contribution of the main architectural components of NST-Net (Section 4.2). In this section, the effect of dataset length is also assessed. For the largest datasets, the available data are truncated to the last 25%, 50%, 75%, and 100% of their original length, corresponding to approximately 2, 5, 7, and 9–10 months, respectively. The splits are recomputed for each truncated dataset to ensure that the training, validation, and test sets remain proportional across all history lengths, rather than retaining fixed portions of the original dataset and thereby changing the relative allocation of data as the available history decreases. Consequently, shorter-history configurations are evaluated on correspondingly shorter test periods rather than on the original held-out period. To characterize the resulting difference between the data available for training and the corresponding test period, the median feature-wise Wasserstein distance (WS) [41] is also reported, with lower values indicating greater distributional similarity.

Quantitative evaluation is particularly challenging because none of the five datasets contains verified anomaly labels. Consequently, detector performance cannot be assessed directly and instead relies on a synthetic anomaly injection protocol applied exclusively to the test set, where representative point, contextual, and collective anomalies are injected in both univariate and multivariate settings. The magnitude of each injected anomaly is controlled by a dimensionless severity parameter, sev, which scales the perturbation relative to the robust feature scale $n_{f}$ defined in Section 3.2.3. Thus, the final perturbation magnitude is $sev\cdot n_{f}$, making the severity feature-adaptive and independent of the original measurement units. This parameter is used for point anomalies and the magnitude-based collective anomalies (shift and variance), whereas contextual and frozen anomalies are generated through changes in temporal context or signal dynamics rather than by directly scaling a perturbation magnitude. The use of these severity levels allows us to evaluate the model under different magnitudes of deviation from the normal behavior of each monitored variable. For instance, sev=0.5 represents smaller deviations, corresponding to anomalies that remain closer to the typical variability of the monitored signal and are therefore more difficult to distinguish from normal fluctuations; sev=1 represents a substantial deviation from the normal signal range; and sev=2 represents a much larger deviation, corresponding to more pronounced abnormal conditions. Anomalies are injected in a round-robin fashion in the order collective, contextual, and point, reflecting their decreasing temporal lengths and ensuring that longer anomaly segments are allocated first. The injection process is constrained by an occupancy budget, while cooldown intervals prevent anomalies from overlapping or occurring too closely together. Consequently, the realized anomaly ratio can differ from the targeted ratio, particularly for short test sets and high target densities. Appendix E shows the algorithm that governs the anomaly injection pipeline.

Since the injected anomalies constitute the only available ground truth, the evaluation further assumes that the remaining test observations are normal. However, the original signals may themselves contain genuine but unlabeled anomalies, which are indistinguishable from normal samples during evaluation and therefore act as noisy negatives. Rather than attempting to identify or remove such events through unverifiable assumptions, they are retained, and threshold-independent ranking metrics are adopted to minimize dependence on arbitrary operating thresholds while providing robustness to this unavoidable label uncertainty. Specifically, Average Precision (AP), Precision@K (P@K), and Recall@K (R@K) are computed with K fixed to 10% of the test-set length. Detections are evaluated pointwise (PW) rather than using point-adjusted (PA) metrics, as PA has been shown to artificially inflate reported performance and favor trivial detectors [8], while PW evaluation better reflects the real-time objective of raising alerts as early as possible. Under this labeling convention, each window inherits the label of the single raw timestep it is anchored to; consequently, the realized proportion of anomalous points and the realized proportion of anomalous windows are numerically identical. Since recent studies have demonstrated that evaluation protocols themselves can substantially influence reported MTSAD performance independently of detector quality [23], Section 4.3 systematically analyzes the impact of the proposed evaluation pipeline, including the effect of anomaly prevalence (2%, 5%, 10%, and 20%), anomaly type (point, contextual, and collective) and severity (sev∈{0.5,1,2}), and the choice of PW against PA evaluation. All results regarding NST-Net correspond to the mean and standard deviation over ten independent runs, i.e., ten seeds.

Fourth, the semantic-rule module is evaluated independently through a set of label-free sanity checks that characterize its intrinsic behavior (Section 4.4). This study includes the activation rate (AR ∈[0,1]), which identifies rules that are either overly permissive or too rare to be operationally meaningful; burstiness (Burst ∈[−1,1]), which measures the temporal clustering of activations and distinguishes cyclic (Burst ≈−1), random (Burst ≈0), and highly structured (Burst ≈1) patterns; and the noise sensitivity index (NSI ∈[0,1]), which quantifies robustness by measuring the fraction of rule outputs that change under small perturbations of the input signal (zero-mean Gaussian noise corresponding to 5% of the standard deviation of each variable, over ten trials). Healthy rules exhibit activation rates in the range [0.01,0.1], intermediate burstiness (0.3–0.6), and low noise sensitivity (≤0.05). These definitions, calculation procedures, and reference ranges are introduced by the authors as descriptive diagnostics for this study and should not be interpreted as universal thresholds for semantic-rule quality. They are intended as evidence of rule stability and robustness rather than as measures of hazard-detection accuracy; empirical validation against confirmed conservation hazards is constrained by the limited availability of documented events across the pilot sites and by the strong site-specificity of deterioration processes, which makes cross-site extrapolation difficult, a barrier we quantify directly in Appendix G by applying each site’s rule set unmodified to the other pilot sites. In addition, the proportion of rules that are regime-testable within each site’s monitoring window is reported, since rules targeting seasonal or infrequent conditions (e.g., freeze–thaw cycling, extreme solar radiation) cannot be meaningfully evaluated if the corresponding regime did not occur during the available period.

Finally, the complementarity between the DL and the semantic module is analyzed to assess the operational value of the parallel-ensemble system (Section 4.5). Three metrics across all sites are reported, including the complementarity index (CI), the median percentile of semantic-flagged timesteps within the DL score distribution, and Cohen’s *d* ($\delta_{\mathrm{Cohen}}$) [42]. CI is defined as the fraction of semantic alarms ($y^{\mathrm{SEM}}=1$) not detected by the DL model ($y^{\mathrm{DL}}=0$). The median percentile indicates whether NST-Net assigns high anomaly scores to semantic events; both CI and this metric are defined by the authors for this study. $\delta_{\mathrm{Cohen}}$ measures the effect size between $s^{\mathrm{DL}}$ for $y^{\mathrm{SEM}}=1$ and $y^{\mathrm{SEM}}=0$, computed within the subset where $y^{\mathrm{DL}}=0$, thereby quantifying the separation independently of sample size. A system with CI ≈1, median percentile ≈0.5, and $\delta_{\mathrm{Cohen}}\approx 0$ indicates that the semantic module identifies structurally distinct anomaly types not captured by the learned model, confirming genuine complementarity rather than redundancy. In addition, representative time series for P1, selected for its perfect complementarity (CI = 1.00), and for P2, selected as the site with the lowest CI, are provided to demonstrate the operational relevance of the system under both complete and partial separation between the two detection mechanisms.

## 4. Results and Discussion

### 4.1. NST-Net: Overall Results

Table 3 reports AP, P@K, and R@K for NST-Net and six representative baselines (IForest [37], USAD [38], GDN [39], MTAD-GAT [18], AnomTR [14], and TimesNet [40]). Across the five pilot sites, the evaluated methods exhibit substantial variability, highlighting the difficulty of AD in real-world CH monitoring data. The absolute metric values should be interpreted in the context of the evaluation setting: the synthetic injection protocol adopted in Section 4.1, Section 4.2 and Section 4.3 emphasizes sharp, short-duration deviations and does not represent slow, cumulative degradation processes, a boundary we quantify directly in Appendix F. Unlike standard MTSAD benchmarks, these datasets are not anomaly-free before injection but contain unlabeled anomalous events; injected anomalies therefore coexist with real, unlabeled anomalies that are not scored as positives, making the ranking task harder and depressing AP, P@K, and R@K for all methods. For a random ranking, expected AP equals the injected-anomaly prevalence. Under the default 5% target injection frequency, this baseline is approximately 0.05; however, the realized prevalence, and therefore the effective random baseline, varies by site and target density, from 0.04 at P2 to 0.12 at P5, as shown in Section 4.3. AP values well above the realized, site-specific baseline indicate meaningful ranking performance despite the harder evaluation setting.

Performance varies substantially across pilot sites, and monitoring duration emerges as the primary explanatory factor. On P1, P3, and P4, the three sites with the longest deployments, NST-Net achieves its most consistent and highest absolute scores. On P2 and P5, which have the shortest monitoring windows, performance degrades for all DL methods, and the variance increases markedly. P5 in particular yields high standard deviations across all metrics, which precludes statistically precise conclusions about method ranking at this site; results should therefore be interpreted with caution and regarded as indicative rather than definitive.

Among the baselines, MTAD-GAT performs most competitively on P1, P3, and P4. This suggests that the graph-based spatiotemporal attention mechanism of MTAD-GAT benefits from the same data richness conditions that favor NST-Net and that on long-duration datasets the gap between DL methods narrows. In contrast, IForest emerges as the strongest baseline on P2 and even the best model on P5. This is consistent with the established finding that classical ensemble methods retain a competitive advantage when training windows are insufficient for deep architectures to converge reliably.

The NST-Net-bp results illuminate the respective contributions of architecture and preprocessing. On P2 and P5, NST-Net-bp outperforms full NST-Net, suggesting that on short deployments with severe missingness the full preprocessing pipeline is counterproductive. Two mechanisms may explain this. First, shorter campaigns exhibit less temporal drift between training and test distributions, reducing the non-stationarity correction that RevIN is specifically designed to address. Second, the alive mask, while beneficial when missingness is informative, may introduce additional optimization difficulty when the fraction of masked timesteps is so large that the effective training signal per window becomes too sparse to guide learning reliably. On P1, P3, and P4, where deployments are longer and missingness is not so high, the pattern reverses: full NST-Net consistently outperforms NST-Net-bp, confirming that RevIN, the alive mask, and cyclic temporal features each contribute positively when data quality and duration are sufficient to exploit them.

Crucially, NST-Net-bp remains highly competitive across the five pilots and outperforms the existing baselines under most conditions. This indicates that the spatiotemporal dual-objective architecture provides a substantial contribution to NST-Net’s performance independently of the preprocessing pipeline. Thus, NST-Net’s overall advantage arises from the joint contribution of its architecture and preprocessing pipeline: both are needed to achieve peak performance on longer, lower-missingness campaigns, whereas on the shortest, most severely missing deployments the architecture alone is sufficient and the additional preprocessing becomes counterproductive.

Table 4 contextualizes NST-Net’s detection advantage in terms of computational cost. NST-Net does not achieve the lowest inference latency: IForest is the fastest method across all sites, and among deep models, USAD and AnomTR are consistently faster, reflecting the overhead of the sequential TCN–attention design. However, NST-Net achieves the lowest memory footprint among all DL methods by a substantial margin, requiring only 14–21 MB across all sites compared to 137–643 MB for the other deep baselines. This advantage is architecturally motivated: USAD employs large encoder–decoder networks; graph- and attention-based methods (GDN, MTAD-GAT, AnomTR) must materialize F×F pairwise interaction matrices at every forward pass, whose memory cost scales quadratically with the number of features; and TimesNet generates large temporal representations during its multi-scale processing, resulting in higher peak memory consumption. In contrast, NST-Net’s single-pass cross-feature attention with a low-dimensional bottleneck keeps memory consumption bounded. NST-Net also ranks second among deep models in parameter count (0.026 M) after GDN (0.004 M). Critically, all methods operate well below the 30 min resampling interval that defines the effective temporal resolution of the monitoring system, meaning that latency differences of a few milliseconds carry no operational consequence for real-time deployment. The efficiency profile of NST-Net, with the lowest deep model memory footprint and second fewest parameters at sub-second latency, therefore directly supports its suitability for continuous real-time deployment in resource-constrained environments, complementing the detection performance advantage established in Table 3.

### 4.2. NST-Net: Ablation Study

The ablation study shown in Table 5 confirms that the improvements achieved by NST-Net arise from the interaction of its architectural components rather than from a single design choice. The complete model achieves the best overall performance on the three longest monitoring campaigns (P1, P3, and P4), while only isolated ablations slightly outperform it on P2 and P5. For these shorter campaigns, the differences between variants are generally comparable to the reported standard deviations, indicating that the contribution of individual modules becomes less stable when limited temporal information is available. Lower values in Table 5 indicate more important architectural components, as their removal results in the largest performance degradation.

The influence of median-based RevIN is strongly site (and time) dependent. The largest improvement is obtained on P1, followed by P3 and P4. Note that the high NS of P4 (Figure 3) refers only to the four variables selected, rather than to the whole dataset, explaining why P4 is not the most affected by RevIN removal. Slight improvements are even obtained by removing RevIN on P2 and for AP on P5. This does not imply that RevIN is unnecessary on these sites; rather, its effect depends on the magnitude of distribution shifts between training and test data. Shorter monitoring periods exhibit greater similarity between training and test distributions due to their temporal proximity, reducing the need for distribution-shift mitigation. At the same time, normalization may attenuate milder anomalous deviations, making RevIN less advantageous overall. This observation is consistent with the better performance of NST-Net-bp compared with NST-Net reported in Table 3.

Among all components, feature-wise attention is the only one whose removal consistently degrades performance across every pilot, making it the most universally beneficial part of the architecture. This reflects the strong coupling between variables, where modeling inter-variable dependencies remains important regardless of monitoring duration or anomaly type. In contrast, the contribution of the TCN depends on the available temporal context. Removing the temporal convolution layers substantially worsens performance on P1, P3, and P4, confirming the importance of multi-scale temporal modeling for long monitoring campaigns. However, the effect is much smaller on P5 and even beneficial on P2, where limited historical data provide less temporal structure to exploit. Together, the results from P2 and P5 suggest that four months of training data (0.8×5 months in P2) are insufficient for reliably exploiting long-range temporal dependencies, and longer datasets are required for effective MTSAD in CH. To directly assess the effect of available training duration, a controlled ablation is performed on the three longest-monitored pilots. Table 6 shows the performance of NST-Net for different dataset lengths, alongside the Wasserstein distance between the training and test distributions.

Overall, the shortest setting (25%, ≈2 months) generally produces the weakest performance, although P3 is an exception and also exhibits substantial variability. Beyond this shortest setting, performance does not improve monotonically with increasing dataset length: P1 performs best at 50%, P4 at 75%, and P3 with the full history. Thus, adding more historical data does not necessarily improve AD performance in these sub-annual campaigns. This behavior reflects a trade-off between temporal coverage and distribution shift. Longer histories provide more observations but also incorporate increasingly distant temporal regimes, which can reduce their relevance to the test period. The increasing Wasserstein distances support this interpretation. Conversely, the shortest histories are more temporally aligned with the test period but may provide insufficient data for robust learning. Overall, the results suggest that NST-Net benefits from additional history up to a site-dependent point, beyond which the benefit of more data can be offset by increasing train–test distribution shift.

**Figure 4:**
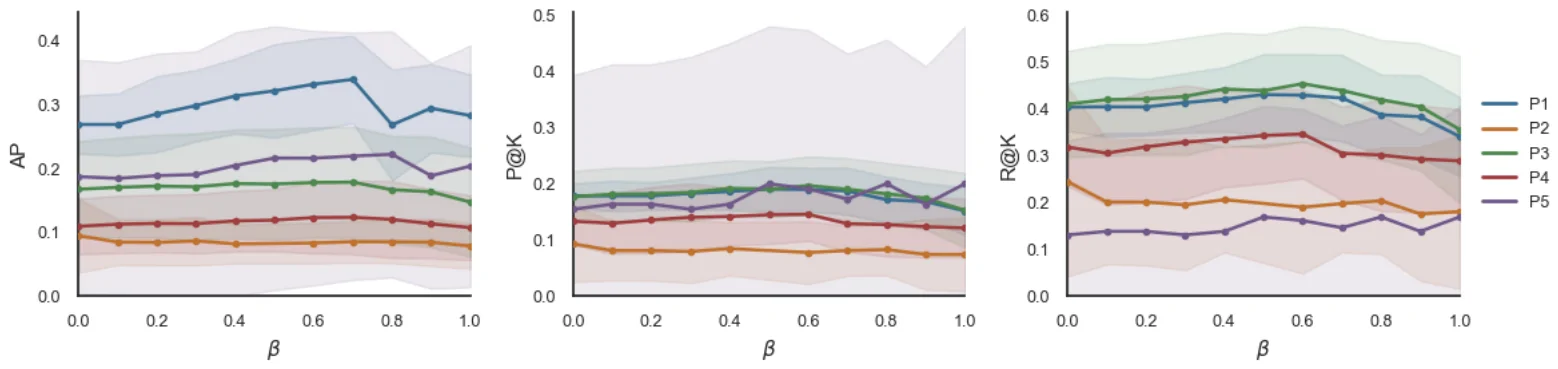
Performance of models as a function of the β parameter, which controls the relative weighting of the reconstruction and forecasting objectives. Solid lines denote the mean over 10 runs, and shaded regions indicate one standard deviation.

The reconstruction and forecasting objectives are complementary, although their relative importance varies across pilot sites. As shown in Table 5, removing either branch generally degrades performance. P1, P3, and P4 benefit from both objectives, whereas P2 performs slightly better without reconstruction and P5 without forecasting. These exceptions suggest that the optimal balance depends on the characteristics of the monitored environment rather than on one objective being universally superior. Figure 4 analyzes the influence of the weighting parameter β. Across all three metrics, mean performance changes only gradually as β varies, indicating that NST-Net is not particularly sensitive to the exact weighting. P1–P4 show a slight performance degradation near the reconstruction-only objective (β=1), whereas P5 benefits from it. However, these differences are small compared with the run-to-run variability, as evidenced by the broad standard deviation bands, particularly for P5. Consequently, no single weighting is consistently optimal, and β=0.5 provides a robust default across all pilot sites.

### 4.3. NST-Net: Evaluation Protocol Sensitivity

Beyond the sensitivity to model configuration and data history examined above, it is also important to assess how robust the reported performance is to the characteristics of the evaluation setup itself. We therefore investigate the effects of anomaly density, anomaly type and severity, and the choice of pointwise (PW) versus point-adjusted (PA) evaluation.

Table 7 reports the realized injected ratios for the five pilot sites under targeted injection levels (2%, 5%, 10%, and 20%), alongside the corresponding detection performance, measured in terms of AP, for the proposed NST-Net model under the default mixed-anomaly injection protocol. Injected ratios are reported at the point level; under the pointwise labeling convention, window-level ratios are identical. For the performance, we report only AP, as it is bounded in [0,1] independently of the number of injected anomalies $N_{a}$. This is a crucial property for a fair comparison across different densities: when $N_{a}<K$ (2%, 5%), Precision@K is capped below 1 by $\min(1,N_{a}/K)$; conversely, when $N_{a}>K$ (20%), Recall@K is capped below 1 by $\min(1,K/N_{a})$. AP avoids both limitations by integrating over all recall thresholds, providing a density-independent measure of ranking quality.

AP increases consistently with the realized injection ratio across all five pilot sites, with the highest AP always corresponding to the highest realized density. This trend is expected given the dependence of AP on positive-class prevalence and should therefore not be interpreted as evidence that the detector becomes intrinsically more effective as more anomalies are injected. Rather, it indicates that AP is sensitive to anomaly prevalence, while the consistent trend across sites suggests that the model’s relative ranking behavior remains stable across different injection densities. This also motivates reporting the realized, rather than only the targeted, injection ratios, as the two can differ substantially under the constraints of the injection procedure. At higher target densities, the realized ratios increasingly fall below the requested values, particularly for P1, P3, and P4. For instance, P1 achieves 1.64%, 4.43%, 7.86%, and 13.58% for targets of 2%, 5%, 10%, and 20%, respectively. This saturation reflects the occupancy and cooldown constraints, which prevent anomalies from being placed too close to previously injected instances, together with the finite test-set length and the availability of suitable candidates for the different anomaly types. P5 exhibits the strongest saturation, with approximately 11.82% realized for targets from 2% to 10%, as a single collective anomaly already accounts for this proportion of the short test period. The realized ratio therefore remains unchanged until the 20% target, where it increases only slightly to 12.73%.

Anomaly density represents only one dimension of the evaluation protocol. To further characterize its sensitivity, we examine NST-Net across different anomaly types and severities and compare pointwise (PW) with point-adjusted (PA) evaluation. Table 8 reports R@K because, at $N_{a}\approx 5\%$ of the test-set length, it has a common upper bound across almost all injection strategies. The only exception is P5, where one collective anomaly exceeds the evaluation budget (K=10%), yielding a theoretical maximum R@K of 0.846 for collective anomalies. R@K is preferred over AP here because it more directly captures segment coverage and therefore the benefit of switching from PW to PA evaluation. Note that Table 3, Table 4, Table 5, Table 6 and Table 7 report results under the default mixed-anomaly injection protocol used throughout this paper, whereas Table 8 isolates individual anomaly types. Consequently, the absolute values are not directly comparable.

Across all pilot sites, R@K increases with anomaly severity regardless of anomaly type. This behavior confirms that the proposed synthetic injection protocol behaves as intended, producing progressively more distinguishable perturbations with an increase in sev. The ranking of anomaly types remains remarkably consistent across the five pilot sites. Point anomalies are detected most reliably, followed by collective variance (Coll. Var.) and shift (Coll. Shf.) anomalies, whereas frozen (Coll. Frz.) and contextual (Cont.) anomalies remain considerably more challenging. This consistency suggests that the observed behavior primarily reflects intrinsic properties of the anomaly classes rather than dataset-specific characteristics. NST-Net performs well on point anomalies, as isolated deviations directly increase both reconstruction and forecasting residuals. In contrast, collective shift anomalies are more difficult because RevIN computes normalization statistics independently within each input window. When a shift occupies a substantial portion of the window, the median is displaced towards the anomalous segment, attenuating the injected deviation before reconstruction or forecasting. Collective variance anomalies are less affected because increased variability does not stabilize the window statistics in the same way. Frozen and contextual anomalies are even less compatible with residual-based detection. Frozen segments remain highly predictable for both reconstruction and forecasting objectives, producing small residuals despite being operationally abnormal. Likewise, contextual anomalies are generated from real in-distribution observations and therefore remain statistically plausible even though their temporal context is incorrect.

PA evaluation consistently improves R@K over PW evaluation because it credits detections occurring anywhere within an anomalous segment. As expected, point anomalies exhibit no improvement (Δ=0), since they occupy a single timestamp. The largest gains are observed for contextual anomalies (up to +0.374 in P4) and collective freezing (up to +0.197 in P1), followed by collective shift and collective variance anomalies. These improvements generally decrease with anomaly severity because higher-severity anomalies are increasingly detected at the exact anomalous timestamps. The magnitude of the PA–PW gap also varies across pilots. P1 exhibits the highest overall R@K together with substantial PA gains, indicating that detections often occur within, but not necessarily at the onset of, anomalous segments. P3 and P4 show similar behavior, with moderate-to-large improvements for collective anomalies. P2 consistently achieves lower R@K, suggesting that anomalies are intrinsically harder to distinguish in this dataset despite still benefiting from PA. In contrast, P5 shows virtually no difference between PA and PW across all anomaly types. Combined with its consistently low R@K, particularly the complete failure to detect contextual anomalies, and high variability, this indicates that the detector fails to reliably detect the anomalous segments altogether. Consequently, PA provides no additional improvements in this dataset.

Overall, these experiments demonstrate that evaluation outcomes depend not only on detector quality but also on the anomaly injection pipeline, dataset characteristics, and the adopted evaluation protocol. Reporting results across multiple anomaly densities, types, severities, pilot sites, and evaluation criteria therefore provides a more realistic assessment of detector robustness than relying on a single benchmark configuration. All anomaly types evaluated here remain short relative to the test-set length; Appendix F extends this evaluation-protocol analysis with a substantially longer, slow-drift anomaly type.

### 4.4. Semantic Layer: Inherent Results

**Figure 5:**
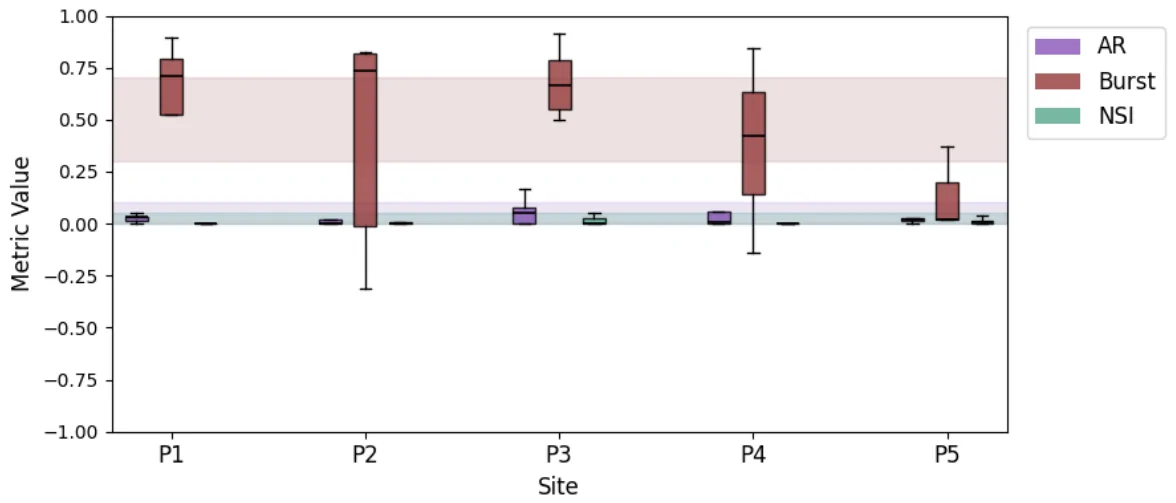
Box plots of the activation rate (AR), burstiness (Burst), and noise sensitivity index (NSI) for all the rules of each pilot site. Background colors matching the metrics report the desired values.

Figure 5 shows the distribution of the activation rate, burstiness, and noise sensitivity index across sites as box plots covering all rules described in Appendix C. Background colors indicate desired values, with box plots falling in these regions as the objective. As emphasized in Section 3.5, these diagnostic metrics characterize the intrinsic stability and robustness of the semantic rules; they are not designed to, and should not be interpreted as, a measure of hazard-detection accuracy, which cannot be assessed here due to the absence of confirmed conservation-event labels.

AR values fall within the expected healthy range for the final rule set, with median values between 0.01 and 0.07, confirming that the rules are neither persistently active nor so rare that they fail to contribute to the detection process. Importantly, very low AR values for specific rules should not be interpreted as underperformance. Several rules are designed to capture seasonal conditions that were not present during the available monitoring period. For instance, rules targeting extreme solar-radiation events in P3 or winter freezing conditions in P5 remain inactive because the corresponding regimes did not occur in the dataset (P3: measurements from autumn to early summer; P5: measurements from spring to early summer). Table 9 summarizes regime testability by site: P3 and P5 show the lowest coverage (90.91% and 66.67%, respectively), while P1, P2, and P4 achieve full regime coverage within the available data.

Burstiness exhibits the largest variability across sites, and this variability is both expected and informative. Median values remain close to the desired range, indicating that most rules activate in coherent temporal episodes rather than isolated spikes. The presence of negative and strongly positive values reflects the heterogeneous nature of the rule set. Negative values correspond to rules driven by cyclic environmental processes, such as soil moisture dynamics or wind, correlated with temperature–humidity daily cycles; this cyclic signature is comparatively more prominent in P2 and P4, producing wider interquartile ranges and lower medians than the other pilots. Conversely, highly positive values correspond to event-driven or accumulation-based rules. This behavior is particularly evident in P3, where several rules monitor progressive accumulation processes and therefore exhibit tightly clustered activation episodes.

Noise sensitivity remains uniformly low across all sites, with values tightly concentrated around zero. This indicates that the rules are robust to minor fluctuations in the sensor signals and do not respond to measurement noise or ambient variability. Low NSI is a critical property for operational deployment: it ensures that rule activations correspond to meaningful environmental deviations rather than artifacts of sensor instability.

Overall, the semantic rules exhibit stable activation patterns, coherent temporal behavior, and low sensitivity to measurement noise across all pilot sites, indicating that the expert knowledge module demonstrates consistent and robust behavior under the observed monitoring regime.

### 4.5. Late-Fusion System Behavior: Quantitative Results and Case Study

Having established that the semantic rules are intrinsically stable and well calibrated, we next evaluate their interaction with the DL detector. The final objective is not to assess the semantic module in isolation but to determine whether it contributes information beyond that already captured by NST-Net. To this end, Table 10 reports the three complementary metrics introduced in Section 3.5.

The results support the complementarity hypothesis on the CI axis but reveal a more nuanced picture on the remaining metrics. CI remains high across all five sites (0.873–1.000), confirming that the large majority of semantic alarms are missed by the DL detector. For P1, the CI of 1.000 corresponds to zero jointly detected alarm timesteps, with all semantic alarms belonging to the semantic-only category. The median percentile and $\delta_{\mathrm{Cohen}}$, however, do not uniformly match the idealized $\left(\mathrm{percentile}\approx 0.5,\;\delta_{\mathrm{Cohen}}\approx 0\right)$ profile. P2 and P4 approximate it closely (percentile 0.550/0.526, $\delta_{\mathrm{Cohen}}=0.171/0.046$), consistent with semantic events that are statistically unremarkable to the DL model in either direction. P1 and P3 instead show a marked negative shift ($\delta_{\mathrm{Cohen}}=-0.559$ and −1.967, respectively), with P1’s median percentile also falling below mid-range (0.337). This indicates that at these sites semantic alarms coincide with windows the DL model scores as particularly normal, rather than merely orthogonal to its scoring axis. This negative-shift pattern is not inconsistent with complementarity but reflects a stronger form of it: rather than semantic and DL alarms being statistically independent, the semantic module at P1 and P3 appears to flag conditions that the reconstruction and/or forecasting objective rewards as easy to predict. P5 exhibits a milder version of the same tendency ($\delta_{\mathrm{Cohen}}=-0.173$, percentile 0.476).

Overall, the results indicate two forms of complementarity: semantic events may be largely unremarkable along the DL scoring axis (P2, P4) or may be systematically associated with low DL anomaly scores (P1, P3, and, to a lesser extent, P5). In both cases, the results show that semantic information provides a complementary signal that cannot be reliably recovered from the DL anomaly score alone, supporting its inclusion in the proposed late-fusion system.

**Figure 6:**
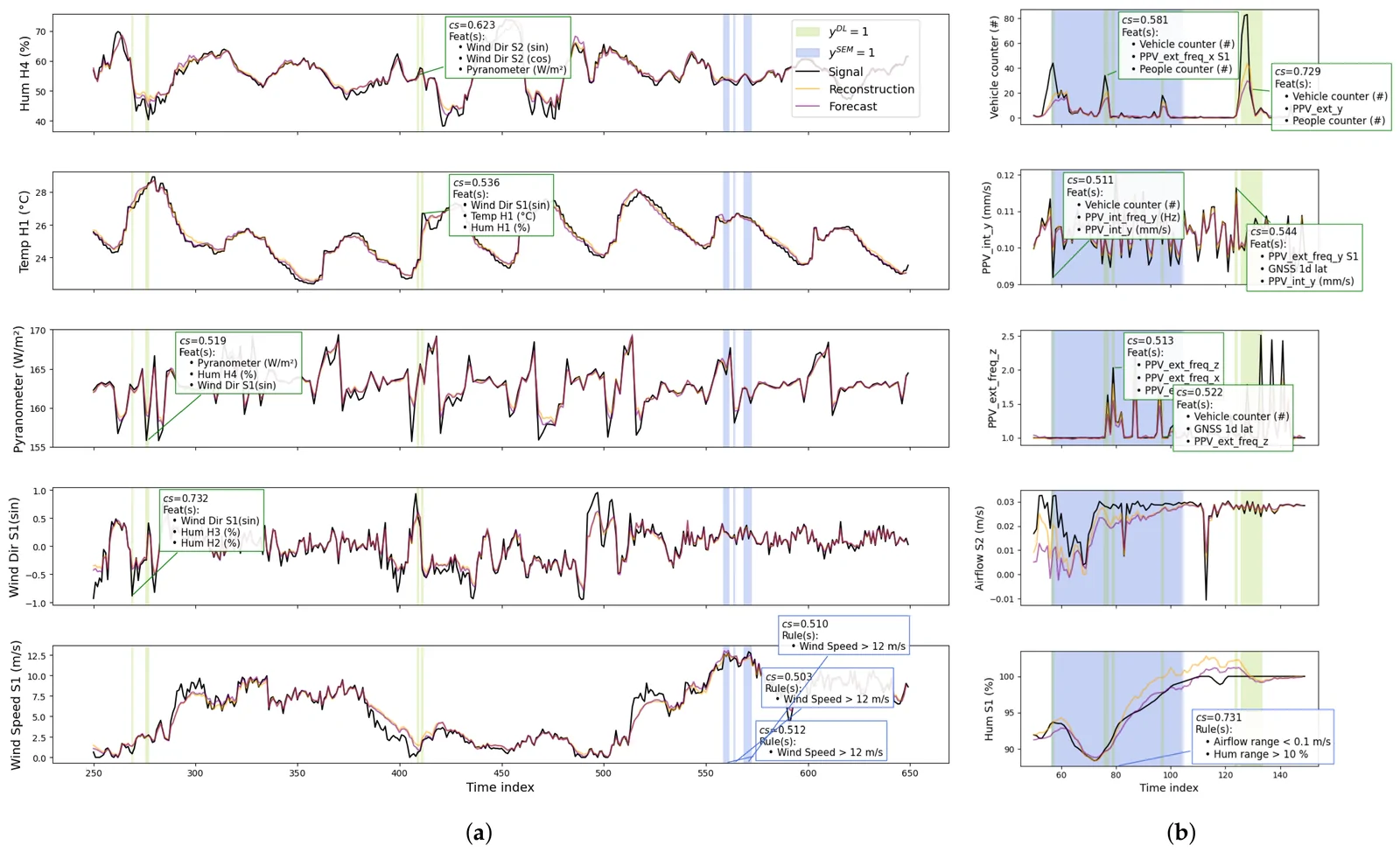
Case studies of (**a**) P1 and (**b**) P2. Original time series (black) are plotted alongside reconstruction (orange) and forecasting (purple) outputs. Green-shaded regions correspond to NST-Net alarms ($y^{\mathrm{DL}}=1$); blue-shaded regions correspond to semantic module alarms ($y^{\mathrm{SEM}}=1$). Confidence scores (cs) and top contributing features are annotated for each DL (green) and SEM (blue) detection.

To illustrate the complementarity results, we present a qualitative case study on P1, selected because it exhibits complete separation (CI = 1.00, Table 10) and provides the clearest illustration of the two complementary detection mechanisms. Figure 6a shows a representative segment of the P1 test period with five variables. The known event date (Table 1) is not shown because it falls within the training period. For comparison, Figure 6b presents a shorter segment from Baltanás (P2), which has the lowest CI among the five pilots (CI=0.873, Table 10). Unlike P1, P2 exhibits greater overlap between the two detection mechanisms, making individual alarms more difficult to distinguish and interpret; the shorter segment therefore focuses on a representative example of this overlap. To maintain simplicity, only the smallest set of variables that explains all the alarms is displayed. Nevertheless, other variables may also contribute to the anomalies observed. Green-shaded regions denote alarms from NST-Net ($y^{\mathrm{DL}}=1$), and blue-shaded regions denote alarms from the semantic module ($y^{\mathrm{SEM}}=1$). Each alarm $y_{t}=1$ is annotated with its confidence score (cs) and the variables (or rules) most responsible for the decision.

In P1, NST-Net flags anomalies in two closely spaced detection pairs within this segment, each driven by elevated reconstruction and forecasting error rather than any semantic rule. The first pair occurs at t≈268–280. The earlier detection is driven by Wind Dir S1(sin) (fourth variable), jointly with Hum H3 and Hum H2, which have similar behaviour to Hum H4 (first variable). In this case, the deviation in Wind Dir leads to a mild cs (cs=0.732). The detection immediately following is driven primarily by a deviation in the Pyranometer signal (third variable plot), together with secondary contributions from Hum H4 and Wind Dir S1(sin). The deviations are relatively small, leading to a low confidence score above the threshold (cs=0.519). A second pair appears at t≈405–410. The first anomaly (cs=0.623) is driven by Wind Dir S2 (sin and cos), with similar behavior to Wind Dir S1 (sin), and the Pyranometer. After that, the last anomaly (cs=0.536) is due to Wind Dir S1 (sin), Temp H1 (second graph), and Hum H1, which shows similar behavior to that of Hum H4. As with the earlier pair, the top contributing features are not confined to a single sensor but propagate across physically coupled channels. None of these four NST-Net detections coincide with a semantic alarm, consistent with the absence of overlap (CI=1.00) reported for this site.

The semantic module activates independently and later in the segment, at t≈560–580, where three closely spaced alarms fire (cs=0.510,0.503,0.512), all triggered by the same rule: wind speed exceeding the 12 m/s threshold (Wind Speed S1, last variable shown). Critically, NST-Net does not flag this period at all: as shown in the bottom panel, reconstruction and forecasting track the rising wind speed closely throughout the approach to and crossing of the threshold, since a sustained, gradual increase toward the upper end of the observed range does not register as a statistical deviation. In fact, reconstruction and forecasting errors are even lower in the semantic regions than in other parts of the variable time series, coinciding with the negative shift observed in Table 10 for P1. This is precisely the converse failure mode the semantic module is designed to catch: an operationally meaningful physical threshold crossing that remains in-distribution from the model’s perspective and therefore produces no reconstruction or forecasting error.

In the shorter P2 segment (Figure 6b), an example of overlap between the DL and semantic modules is shown. In this example, NST-Net alarms are driven primarily by anthropogenic and vibration-related variables (Vehicle counter, People counter, and the PPV vibration features), consistent with transient site activity propagating into physically coupled vibration channels. The semantic module, in contrast, activates over a longer window based on P2’s airflow and humidity daily-range rules (Appendix C). Unlike P1, the two types of alarms partially overlap: NST-Net detections occur both at the edges of and within the semantic-alarm window. Nevertheless, most of the semantic window remains undetected by NST-Net, while additional DL detections occur after the semantic window has ended without a corresponding semantic-rule activation.

To extend this complementarity evidence beyond P1 and P2, we briefly summarize the alarm interactions observed at the remaining pilot sites, restricted to the test period. At P3, semantic alarms show only 3.9% overlap with NST-Net detections, driven mainly by the visitor-count rule together with the rapid humidity-change and cumulative 7-day rainfall rules; this near-complete separation is closer to the pattern observed at P1, consistent with the strongly negative Cohen’s *d* reported for this site in Table 10. At P4, semantic alarms, driven mainly by the 7-day cumulative rainfall rule, show 9.5% overlap with NST-Net detections, more comparable to the partial overlap observed at P2. At P5, alarms mainly driven by the vibration-based rules show 12.6% overlap with NST-Net detections; while numerically the largest overlap among P3–P5, the mildly negative Cohen’s *d* for this site (Table 10) still places it closer to the negative-shift regime of P1/P3 than to the orthogonal profile of P2/P4. The shorter monitoring duration explains the greater relative overlap and warrants some caution in the interpretation of the results.

The case study illustrates that the DL and semantic components can produce overlapping alarms, as observed for P2, while still capturing different aspects of anomalous behavior. NST-Net identifies distributional deviations across the monitored variables, whereas the semantic module highlights operationally meaningful states captured by the expert rules. Thus, even when their detections coincide, the two components provide complementary information and different interpretative perspectives. This supports the use of a parallel ensemble architecture, allowing both data-driven and knowledge-based mechanisms to contribute to AD in CH monitoring.

## 5. Limitations

Several limitations should be considered when interpreting these results.

First, verified anomaly labels are unavailable for all five pilot sites. The quantitative DL model evaluation therefore relies on controlled synthetic anomaly injection, which provides a reproducible means of assessing detection performance but cannot fully represent real conservation hazards. The results should consequently be interpreted as evidence of performance under the defined synthetic scenarios rather than as direct validation against real-world anomalies. Similarly, the semantic-rule results should be interpreted as operational assessments of rule activation and stability rather than as evidence of confirmed hazard detection, since no sufficiently documented conservation events were available to establish correspondence between rule activations and actual conservation hazards.

Second, none of the five monitoring campaigns covers a complete annual cycle. This limited temporal coverage, together with the strong seasonal variability across the monitoring sites, motivated the use of median-based RevIN, which normalizes each lookback window independently and is therefore designed to absorb low-frequency, within-window shifts. This choice improves robustness to seasonality and heterogeneous operating conditions but, as a side effect, can also attenuate slowly evolving changes in absolute level that may themselves be informative for certain degradation processes; we return to this trade-off for the synthetic injection protocol below. The incomplete annual coverage additionally means that some seasonal semantic rules, such as those targeting solar-radiation peaks at P3 or freeze–thaw conditions at P5, could not be fully exercised: only 27 of 29 rules (93.10%) were exposed to their intended environmental regime, so a low activation rate for the remaining rules should be read as an artifact of missing regime exposure rather than a deficiency of the rule itself.

Third, the synthetic injection protocol focuses mainly on relatively sharp or short-duration deviations and does not assess slow, cumulative degradation processes such as cracking or moisture migration. We provide an initial, quantitative characterization of this boundary in Appendix F, where a slow cumulative-drift anomaly type is injected and evaluated using a prevalence-normalized metric. NST-Net’s ranking performance on this anomaly type falls close to random at most sites, confirming that its detection advantage is concentrated on the sharp, short-duration deviations evaluated in this paper. This is partly a consequence of the available monitoring duration, particularly at P2 and P5, which limits the anomaly length, and partly of the window-local RevIN normalization discussed above, which is not designed to preserve slowly evolving absolute levels. The protocol also imposes practical constraints on the range of anomaly densities that can be evaluated: because injected instances are separated by an occupancy/cooldown mechanism and allocated across collective, contextual, and point anomalies in round-robin fashion, the realized anomaly ratio depends on test-set length, anomaly duration, and candidate availability. Short campaigns in particular can impose a relatively high minimum realized ratio, since the protocol enforces at least one collective instance while cooldown constraints cap the maximum number of non-overlapping instances. The targeted anomaly ratio should therefore be read as a requested injection level rather than an exact realized prevalence. Moreover, the shorter monitoring campaigns, particularly P5, provide fewer injected anomalies and therefore produce more variable performance estimates. These results should consequently be interpreted as indicative rather than statistically precise.

Fourth, a separate limitation concerns the representation of sensor availability and data quality. The alive mask is designed primarily to handle sensor outages and is therefore less sensitive to gradual sensor degradation or drift. In addition, the 24 h interpolation tolerance used during resampling, introduced to accommodate the daily-cadence T/H sensors at P2, may attenuate genuine physical changes occurring entirely within an unobserved interval. This interpolation does not affect sensor availability, which is governed independently by the alive mask, but it can nevertheless smooth changes that occur between observations and are therefore not explicitly represented in the input data.

Fifth, the comparison with state-of-the-art baselines does not completely isolate the contribution of the NST-Net architecture. The baselines could not be modified to incorporate the alive mask, RevIN, or the additional temporal-context features used by NST-Net; their missing observations were instead handled using the common baseline preprocessing. All models were nevertheless trained under the same early-stopping criterion and with hyperparameters similar to those of NST-Net. To assess the influence of preprocessing, we additionally report NST-Net-bp, which uses the same preprocessing as the baselines.

Sixth, the semantic module remains predominantly single-variable. Conservation hazards rarely arise from a single variable exceeding a fixed threshold; they typically emerge from interactions among environmental and structural variables. Only one rule across the five pilot sites, the cumulative freeze–thaw indicator at P3 and P5, currently uses multivariate activation. This demonstrates that multivariate rules are supported by the framework, but they have not yet been systematically designed across sites. Many conservation processes evolve gradually and could benefit from continuous or probabilistic risk measures, but developing and validating such multivariate rules requires substantial domain expertise and sufficiently long, seasonally representative monitoring records, which are limited in the present pilot campaigns. The predominance of interpretable expert-defined thresholds should consequently be regarded as a practical limitation of the present study rather than as an assumption that conservation risk is intrinsically univariate or deterministic.

Finally, the current rule base remains site-specific and must be adapted by conservation experts for each new deployment, limiting the scalability and transferability of the late-fusion framework. We quantify this transfer barrier directly in Appendix G, where each site’s rule set is applied unmodified to the other four pilot sites, showing that rule thresholds do not generalize across sites without site-specific recalibration, thereby limiting transferability. This is an intentional characteristic of the semantic component: the rules encode site-specific physical and conservation knowledge, including expected environmental and structural behavior and relevant thresholds. Because environmental conditions, structural characteristics, and sensor configurations may differ substantially between sites, a condition that constitutes a meaningful deviation at one site cannot necessarily be transferred unchanged to another. The site-specific formulation therefore prioritizes physical interpretability and expert knowledge over a universal rule set, at the cost of requiring expert review and adaptation for new deployments. Developing more flexible and transferable semantic knowledge bases therefore remains an important challenge for the practical deployment of late-fusion AD systems in CH.

## 6. Conclusions

This paper presented a late-fusion framework for real-time, fully unsupervised anomaly detection in cultural heritage monitoring, combining NST-Net, a nested spatiotemporal detector, with a complementary semantic module encoding site-specific expert conservation rules. Evaluated across five real-world datasets, NST-Net outperformed six state-of-the-art baselines on the majority of sites, with the largest margins on the longest-monitored campaigns. This advantage was demonstrated primarily on sharp, short-duration synthetic anomalies rather than on slow, cumulative degradation processes. A matched-preprocessing variant, NST-Net-bp, showed that on the three longest, lowest-missingness campaigns (P1, P3, P4), both the nested spatiotemporal architecture and the full preprocessing pipeline (RevIN, alive mask, cyclic features) contribute jointly to NST-Net’s advantage, whereas on the shortest, most severely missing deployments (P2, P5), the additional preprocessing pipeline is counterproductive. Efficiency profiling further showed that NST-Net achieves the lowest memory footprint of all deep learning baselines (14–21 MB vs. 137–643 MB) at sub-4 ms latency, confirming its suitability for real-time, resource-constrained deployment. Ablation and duration-controlled experiments (Section 4.2) confirmed that feature-wise attention contributes universally, while temporal convolution and RevIN-based normalization require sufficient training history to be reliably beneficial, and that performance does not improve monotonically with additional historical data once train–test distributional drift is taken into account. The reconstruction and forecasting objectives were shown to be complementary but largely interchangeable, with performance stable across a broad range of their relative weighting. Because none of the five deployments contains verified anomaly labels, evaluation relied on a controlled synthetic injection protocol, which further showed that reported detection performance rises with anomaly density (2–20%), as expected from AP’s dependence on prevalence, and is highly sensitive to anomaly type and to the choice between pointwise and point-adjusted scoring, motivating our use of pointwise metrics as consistent with real-time, low-latency alerting. The semantic module exhibited stable behavior in the label-free sanity checks and showed consistently high complementarity with NST-Net, flagging conservation-relevant events that were either statistically orthogonal to or systematically underscored by the deep detector. This supports the use of parallel statistical–semantic detection, as the two components identify complementary forms of anomalous behavior that are not reliably captured by either mechanism alone.

Future work will focus on five complementary directions. First, extending the monitoring campaigns and increasing the amount of available data across the ARGUS pilot sites will enable more reliable evaluation and allow sequence models to learn richer long-term temporal patterns. Second, as larger datasets become available, the synthetic injection protocol will be extended with a monotonic cumulative-trend archetype for direct evaluation of NST-Net’s sensitivity to slow degradation processes, and alternatives to RevIN that explicitly model long-term distributional changes will be investigated. Third, the availability mechanism will be extended to detect gradual sensor degradation and slow drift in addition to complete sensor outages. Fourth, the semantic module will be enhanced by replacing deterministic single-variable rules with probabilistic or fuzzy multivariable rules. Finally, the proposed framework will be deployed across the five ARGUS pilot sites for continuous real-time monitoring, enabling expert feedback to validate detections, refine both the statistical and semantic components, and assess the system’s impact on conservation decision-making.

## Figures and Tables

**Table 1:** Semantic-rule set for pilot site P1. This example illustrates how site-specific expert knowledge is encoded into the proposed semantic module.

Variable	Rule	Category	Score
Rain	>10 mm/h	Point	Equation (11)
Rain	>20 mm in 3 h	Cumulative	Equation (11)
Humidity	>9 crossings around 75% within 3 days	Persistence	Equation (11)
Wind	>12 m/s	Point	Equation (11)
Known dates	9 December 2025 (structural damage)	Known event	Equation (10)

**Table 2:** Summary statistics of the datasets for each pilot site after preprocessing. Reported metrics include the number (#) of variables at each preprocessing stage (Raw, Clean, and DL-ready), variable categories (environmental, structural, and anthropogenic), acquisition duration, native sampling intervals, sample counts before and after resampling to a common 30 min grid, and the proportion of valid observations (alive ratio).

Site	#Vars Raw	#Vars Clean	#Vars DL	Env/Str/Ant Clean (DL)	Duration	Native Sampling (Range, Predom.)	#Samples Raw (≈)	#Samples Clean	Alive Ratio
P1	65	56	49	35 (33)/20 (16)/1 (0)	9 mo	[1 min, 1 h], 1 h	726 K	12,384	0.705
P2	80	62	58	22 (18)/36/4	5 mo ^1^	[5 min, 1 d], 5 min	403 K	6576	0.694
P3	44	42	39	15 (13)/23 (22)/4	9 mo	[5 min, 1 h], 5 min	726 K	13,104	0.704
P4	40	40	33	23 (22)/17 (11)/0	10 mo	[5 min, 1 h], 1 h	806 K	14,016	0.750
P5	102	32	31	10/22 (21)/0	2 mo	[1 min, 30 min], 30 min	11 K	1920	0.647

^1^ All sites span up to 30 June except P2, where restoration works started on 15 April.

**Table 3:** Performance comparison across the five pilot sites. Values are reported as mean ± standard deviation over 10 runs. Arrows indicate the desired values. Bounds of each metric are also indicated. Best values are in bold, and second-best values are underlined.

Method	P1			P2			P3			P4			P5		
	AP↑	P@K↑	R@K↑	AP↑	P@K↑	R@K↑	AP↑	P@K↑	R@K↑	AP↑	P@K↑	R@K↑	AP↑	P@K↑	R@K↑
	[0, 1]	[0, 0.474]	[0, 1]	[0, 1]	[0, 0.586]	[0, 1]	[0, 1]	[0, 0.447]	[0, 1]	[0, 1]	[0, 0.447]	[0, 1]	[0, 1]	[0, 1]	[0, 0.846]
IForest	0.060 ±0.036	0.054 ±0.037	0.125 ±0.080	0.076 ±0.063	0.058 ±0.065	0.162 ±0.187	0.065 ±0.029	0.059 ±0.037	0.137 ±0.098	0.036 ±0.005	0.028 ±0.025	0.068 ±0.059	**0.263** ±0.192	**0.227** ±0.298	**0.192** ±0.252
USAD	0.051 ±0.012	0.046 ±0.046	0.110 ±0.110	0.051 ±0.023	0.000 ±0.000	0.000 ±0.000	0.056 ±0.020	0.051 ±0.045	0.124 ±0.120	0.052 ±0.025	0.060 ±0.048	0.134 ±0.106	0.074 ±0.007	0.000 ±0.000	0.000 ±0.000
GDN	0.093 ±0.082	0.080 ±0.047	0.185 ±0.109	0.043 ±0.017	0.015 ±0.022	0.035 ±0.057	0.079 ±0.039	0.079 ±0.041	0.182 ±0.098	0.058 ±0.022	0.064 ±0.037	0.150 ±0.079	0.166 ±0.115	0.127 ±0.167	0.108 ±0.141
MTAD-GAT	0.216 ±0.094	0.142 ±0.050	0.323 ±0.116	0.057 ±0.047	0.009 ±0.016	0.022 ±0.043	0.088 ±0.038	0.087 ±0.052	0.160 ±0.134	0.070 ±0.035	0.090 ±0.050	0.212 ±0.108	0.122 ±0.096	0.109 ±0.195	0.092 ±0.165
AnomTR	0.046 ±0.006	0.045 ±0.035	0.101 ±0.072	0.048 ±0.032	0.036 ±0.043	0.073 ±0.089	0.046 ±0.006	0.043 ±0.016	0.100 ±0.038	0.043 ±0.009	0.030 ±0.033	0.069 ±0.073	0.153 ±0.118	0.073 ±0.134	0.062 ±0.114
TimesNet	0.065 ±0.011	0.079 ±0.026	0.186 ±0.068	0.053 ±0.018	0.000 ±0.000	0.000 ±0.000	0.070 ±0.024	0.057 ±0.040	0.136 ±0.096	0.060 ±0.024	0.066 ±0.042	0.152 ±0.090	0.071 ±0.010	0.000 ±0.000	0.000 ±0.000
**NST-Net-bp**	0.098 ±0.048	0.087 ±0.045	0.208 ±0.115	**0.097** ±0.066	**0.095** ±0.095	**0.219** ±0.221	0.123 ±0.071	0.109 ±0.056	0.257 ±0.132	0.098 ±0.046	0.109 ±0.066	0.258 ±0.145	0.258 ±0.128	0.218 ±0.288	0.184 ±0.248
**NST-Net**	**0.322** ±0.073	**0.191** ±0.040	**0.431** ±0.086	0.082 ±0.031	0.083 ±0.059	0.199 ±0.136	**0.176** ±0.087	**0.190** ±0.050	**0.439** ±0.121	**0.119** ±0.049	**0.145** ±0.052	**0.343** ±0.102	0.217 ±0.107	0.200 ±0.280	0.169 ±0.237

**Table 4:** Computational efficiency comparison across the five pilot sites. Par. denotes the number of trainable parameters, Lat. denotes anomaly-score inference time for a single input window, and Mem. denotes peak memory consumption. Arrows indicate the desired direction. Best values are in bold and second-best values are underlined.

Method	P1			P2			P3			P4			P5		
	Par.↓	Lat.↓	Mem.↓	Par.↓	Lat.↓	Mem.↓	Par.↓	Lat.↓	Mem.↓	Par.↓	Lat.↓	Mem.↓	Par.↓	Lat.↓	Mem.↓
	(M)	(ms)	(MB)	(M)	(ms)	(MB)	(M)	(ms)	(MB)	(M)	(ms)	(MB)	(M)	(ms)	(MB)
IForest	–	**0.945**	**0.012**	–	**0.371**	**0.008**	–	**0.695**	**0.055**	–	**0.304**	**0.004**	–	**0.101**	**0.004**
USAD	29.451	1.006	282.006	39.934	1.982	642.711	19.651	1.396	200.687	13.210	1.139	321.166	13.210	1.216	137.641
**GDN**	**0.004**	2.032	282.448	**0.004**	1.715	377.507	**0.004**	2.025	200.947	**0.004**	1.164	137.098	**0.004**	2.012	137.909
MTAD-GAT	0.363	1.775	294.173	0.390	1.622	389.359	0.336	1.718	212.534	0.316	1.750	148.924	0.316	1.634	149.734
AnomTR	1.685	1.443	312.817	1.703	1.331	408.700	1.664	1.497	230.826	1.648	1.541	167.154	1.648	1.333	168.910
TimesNet	0.078	2.373	286.187	0.080	2.587	381.370	0.077	2.413	204.556	0.076	2.579	140.618	0.076	2.626	141.447
**NST-Net**	0.026	3.231	18.290	0.026	3.966	21.251	0.026	3.009	16.025	0.026	2.709	14.410	0.026	2.724	14.410

**Table 5:** Ablation study of NST-Net across the five pilot sites. Values are reported as mean ± standard deviation over 10 runs. Arrows indicate the desired values. Bounds of each metric are also indicated. Best values are in bold, and second-best values are underlined.

Variant	P1			P2			P3			P4			P5		
	AP↑	P@K↑	R@K↑	AP↑	P@K↑	R@K↑	AP↑	P@K↑	R@K↑	AP↑	P@K↑	R@K↑	AP↑	P@K↑	R@K↑
	[0, 1]	[0, 0.474]	[0, 1]	[0, 1]	[0, 0.586]	[0, 1]	[0, 1]	[0, 0.447]	[0, 1]	[0, 1]	[0, 0.447]	[0, 1]	[0, 1]	[0, 1]	[0,0.846]
**NST-Net**	**0.322** ±0.073	**0.191** ±0.040	**0.431** ±0.086	0.082 ±0.031	0.083 ±0.059	0.199 ±0.136	**0.176** ±0.087	**0.190** ±0.050	**0.439** ±0.121	**0.119** ±0.049	**0.145** ±0.052	**0.343** ±0.102	0.217 ±0.107	**0.200** ±0.280	**0.169** ±0.237
**– RevIN-med**	0.128 ±0.081	0.115 ±0.071	0.264 ±0.169	0.087 ±0.051	**0.110** ±0.091	0.259 ±0.199	0.160 ±0.088	0.178 ±0.060	0.414 ±0.139	0.116 ±0.057	0.133 ±0.054	0.316 ±0.114	**0.221** ±0.173	0.164 ±0.238	0.139 ±0.201
**– Attention**	0.098 ±0.067	0.090 ±0.058	0.207 ±0.136	0.057 ±0.030	0.035 ±0.055	0.092 ±0.152	0.098 ±0.047	0.070 ±0.052	0.166 ±0.122	0.096 ±0.059	0.086 ±0.049	0.206 ±0.111	0.118 ±0.101	0.064 ±0.136	0.054 ±0.115
**– TCN**	0.130 ±0.104	0.091 ±0.068	0.210 ±0.159	**0.096** ±0.067	0.098 ±0.073	**0.262** ±0.222	0.098 ±0.052	0.081 ±0.050	0.190 ±0.115	0.105 ±0.063	0.112 ±0.041	0.269 ±0.089	0.150 ±0.155	0.100 ±0.237	0.085 ±0.200
**– Reconstruction**	0.269 ±0.046	0.178 ±0.022	0.404 ±0.051	0.095 ±0.058	0.093 ±0.068	0.245 ±0.202	0.168 ±0.076	0.177 ±0.046	0.411 ±0.114	0.109 ±0.044	0.133 ±0.042	0.319 ±0.084	0.187 ±0.183	0.155 ±0.239	0.121 ±0.202
**– Forecast**	0.283 ±0.065	0.152 ±0.043	0.342 ±0.084	0.079 ±0.036	0.074 ±0.066	0.181 ±0.165	0.148 ±0.086	0.153 ±0.066	0.357 ±0.157	0.107 ±0.051	0.121 ±0.052	0.289 ±0.110	0.204 ±0.190	**0.200** ±0.280	**0.169** ±0.237

**Table 6:** Performance study of NST-Net under different dataset durations for P1, P3, and P4. Values are reported as mean ± standard deviation over 10 runs. Arrows indicate the desired values. Bounds of each metric are also indicated. Best values are in bold, and second-best values are underlined. WS denotes the median feature-wise Wasserstein distance between the training and test distributions.

Length	P1				P3				P4			
	AP↑	P@K↑	R@K↑	WS	AP↑	P@K↑	R@K↑	WS	AP↑	P@K↑	R@K↑	WS
	[0, 1]	[0, 0.474]	[0, 1]	-	[0, 1]	[0, 0.447]	[0, 1]	-	[0, 1]	[0, 0.447]	[0, 1]	-
**25%** (≈2 mo)	0.080 ±0.044	0.074 ±0.107	0.131 ±0.189	0.049	**0.292** ±0.388	0.152 ±0.210	0.292 ±0.404	0.033	0.067 ±0.039	0.052 ±0.091	0.082 ±0.145	0.098
**50%** (≈5 mo)	**0.346** ±0.246	**0.209** ±0.124	**0.445** ±0.254	0.185	0.139 ±0.086	0.138 ±0.093	0.298 ±0.168	0.205	0.089 ±0.047	0.094 ±0.072	0.206 ±0.160	0.165
**75%** (≈7 mo)	0.279 ±0.182	0.164 ±0.091	0.370 ±0.200	0.349	0.126 ±0.057	0.154 ±0.049	0.344 ±0.106	0.309	**0.160** ±0.050	**0.183** ±0.051	**0.405** ±0.056	0.221
**100%** (9–10 mo)	0.322 ±0.073	0.191 ±0.040	0.431 ±0.086	0.533	0.176 ±0.087	**0.190** ±0.050	**0.439** ±0.121	0.299	0.119 ±0.049	0.145 ±0.052	0.343 ±0.102	0.427

**Table 7:** Realized injected ratios and performance in terms of AP for different targeted anomaly ratios across the five pilot sites. Arrows indicate the desired values.

	P1		P2		P3		P4		P5	
Targeted	Injected	AP↑	Injected	AP↑	Injected	AP↑	Injected	AP↑	Injected	AP↑
2.00%	1.64%	0.258 ±0.241	2.24%	0.053 ±0.039	2.60%	0.139 ±0.113	2.42%	0.099 ±0.059	11.82%	0.217 ±0.107
5.00%	4.43%	0.322 ±0.073	4.07%	0.082 ±0.031	4.36%	0.176 ±0.087	4.16%	0.119 ±0.049	11.82%	0.217 ±0.107
10.00%	7.86%	0.357 ±0.081	8.17%	0.116 ±0.052	8.72%	0.236 ±0.081	8.11%	0.193 ±0.025	11.82%	0.217 ±0.107
20.00%	13.58%	0.490 ±0.046	13.48%	0.230 ±0.043	13.55%	0.374 ±0.110	13.92%	0.310 ±0.032	12.73%	0.241 ±0.060

**Table 8:** R@K under pointwise (PW) and point-adjusted (PA) evaluation across anomaly types and severities. Δ denotes the absolute improvement obtained by point adjustment ($R@K_{\mathrm{PA}}-R@K_{\mathrm{PW}}$).

		P1			P2			P3			P4			P5		
Type	Sev	PW	PA	Δ	PW	PA	Δ	PW	PA	Δ	PW	PA	Δ	PW	PA	Δ
Point	0.5	0.443 ±0.116	0.443 ±0.116	0.000	0.155 ±0.156	0.155 ±0.156	0.000	0.307 ±0.127	0.307 ±0.127	0.000	0.236 ±0.083	0.236 ±0.083	0.000	0.050 ±0.158	0.050 ±0.158	0.000
	1	0.783 ±0.063	0.783 ±0.063	0.000	0.273 ±0.178	0.273 ±0.178	0.000	0.645 ±0.098	0.645 ±0.098	0.000	0.623 ±0.136	0.623 ±0.136	0.000	0.250 ±0.354	0.250 ±0.354	0.000
	2	0.958 ±0.051	0.958 ±0.051	0.000	0.560 ±0.189	0.560 ±0.189	0.000	0.847 ±0.081	0.847 ±0.081	0.000	0.860 ±0.116	0.860 ±0.116	0.000	0.550 ±0.439	0.550 ±0.439	0.000
Coll. Shf.	0.5	0.311 ±0.086	0.451 ±0.100	0.140	0.120 ±0.097	0.237 ±0.175	0.117	0.219 ±0.069	0.296 ±0.068	0.077	0.183 ±0.050	0.298 ±0.092	0.115	0.139 ±0.220	0.139 ±0.220	0.000
	1	0.585 ±0.107	0.611 ±0.094	0.026	0.226 ±0.112	0.355 ±0.182	0.129	0.457 ±0.065	0.508 ±0.105	0.051	0.453 ±0.067	0.578 ±0.091	0.125	0.215 ±0.214	0.215 ±0.214	0.000
	2	0.793 ±0.099	0.793 ±0.099	0.000	0.451 ±0.133	0.603 ±0.143	0.152	0.711 ±0.052	0.723 ±0.068	0.012	0.660 ±0.068	0.765 ±0.091	0.105	0.362 ±0.185	0.362 ±0.185	0.000
Coll. Var.	0.5	0.479 ±0.077	0.541 ±0.075	0.062	0.167 ±0.083	0.321 ±0.179	0.154	0.404 ±0.099	0.478 ±0.086	0.074	0.355 ±0.058	0.415 ±0.075	0.060	0.146 ±0.234	0.146 ±0.234	0.000
	1	0.764 ±0.095	0.795 ±0.056	0.031	0.307 ±0.109	0.478 ±0.129	0.171	0.625 ±0.091	0.661 ±0.097	0.036	0.577 ±0.058	0.597 ±0.039	0.020	0.239 ±0.203	0.239 ±0.203	0.000
	2	0.920 ±0.061	0.920 ±0.061	0.000	0.555 ±0.081	0.584 ±0.132	0.029	0.790 ±0.062	0.790 ±0.062	0.000	0.763 ±0.051	0.763 ±0.051	0.000	0.362 ±0.162	0.362 ±0.162	0.000
Coll. Frz.	–	0.127 ±0.074	0.324 ±0.189	0.197	0.076 ±0.065	0.185 ±0.128	0.109	0.104 ±0.078	0.249 ±0.146	0.145	0.082 ±0.060	0.209 ±0.133	0.127	0.100 ±0.178	0.100 ±0.178	0.000
Cont.	–	0.129 ±0.098	0.450 ±0.263	0.321	0.050 ±0.043	0.150 ±0.129	0.100	0.246 ±0.097	0.523 ±0.162	0.277	0.093 ±0.071	0.467 ±0.348	0.374	0.000 ±0.000	0.000 ±0.000	0.000

**Table 9:** Summary of expert rules that are regime-testable within each site’s monitored period.

Pilot	Total Rules	Regime-Untestable	% Testable	Untestable Rule(s)
P1	5	0	100%	–
P2	6	0	100%	–
P3	11	1	90.91%	Solar-radiation peaks
P4	4	0	100%	–
P5	3	1	66.67%	Freeze–thaw
Total	29	2	93.10%	–

**Table 10:** Complementarity and statistical distinctiveness of the semantic module. Arrows indicate the desired values.

Pilot	CI ↑	Median Percentile	$\delta_{\mathrm{Cohen}}$
P1	1.000	0.337	−0.559
P2	0.873	0.550	0.171
P3	0.967	0.496	−1.967
P4	0.880	0.526	0.046
P5	0.932	0.476	−0.173

**Figure A1:**
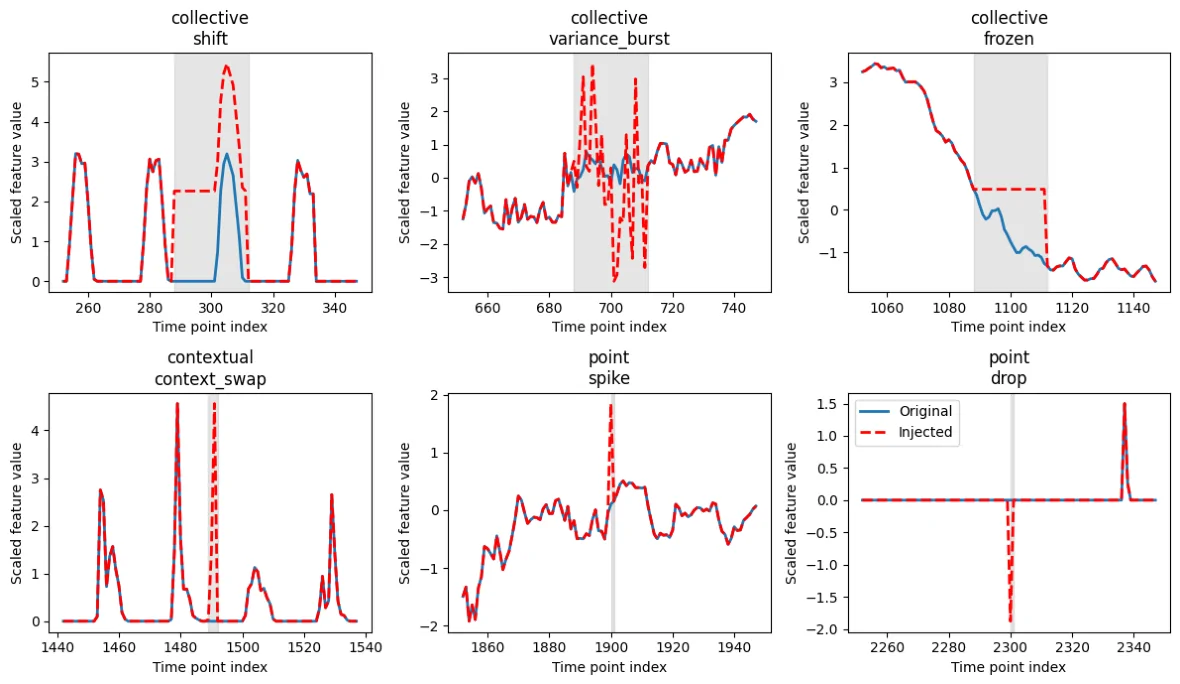
Examples of six types of synthetic anomalies injected across different features. Gray-shaded areas indicate the anomaly injection windows, while blue and dashed red lines denote the original and modified signals, respectively.

## Data Availability

The datasets presented in this article are not readily available because the data are part of an ongoing study. Requests to access the datasets should be directed to the corresponding author. The source code supporting the findings of this study is openly available on the following repository: http://hdl.handle.net/10261/441000 (accessed on 15 September 2026).

## Abbreviations

The following abbreviations are used in this manuscript:

CH	Cultural heritage
WSN	Wireless Sensor Network
AD	Anomaly detection
MTSAD	Multivariate time series anomaly detection
DL	Deep learning
TCN	Temporal Convolutional Network
PA	Point-adjusted
PW	Pointwise
RevIN	Reversible Instance Normalization
EMA	Exponential Moving Average
MAD	Median absolute deviation
ELU	Exponential Linear Unit
RMSNorm	Root Mean Square Layer Normalization

## Appendix A

**Table A1:** Summary of notation used throughout this manuscript, grouped by section.

Symbol	Description	Section
*N*	Number of timestamps in the full multivariate time series	Section 3.1
*F*	Number of sensor variables	Section 3.1
*T*	Lookback window length	Section 3.1
$X\in R^{N\times F}$	Complete multivariate time series	Section 3.1
$x_{t}\in R^{T\times F}$	Input window ending at time *t*	Section 3.1
$s_{t}\geq 0$	Continuous anomaly score for window *t*	Section 3.1
$y_{t}\in \left\{0,1\right\}$	Final binary anomaly label for window *t*	Section 3.1
$cs_{t}\geq 0$	Confidence score, defined only when $y_{t}=1$	Section 3.1
*B*	Batch size	Section 3.2.1
$F_{c}=6$	Number of temporal context features	Section 3.2.1
$F_{\mathrm{ext}}=F+F_{c}$	Total number of input variables	Section 3.2.1
H=32	Latent/hidden representation dimension	Section 3.2.1
$H_{\mathrm{bn}}=16$	Bottleneck dimension of the output decoders	Section 3.2.1
d∈{1,2,4,8}	Dilation factor of the nested spatiotemporal encoder	Section 3.2.1
k=5	Temporal convolution kernel size	Section 3.2.1
$x\in R^{B\times T\times F_{\mathrm{ext}}}$	Input tensor to the encoder	Section 3.2.1
${\hat{x}}^{\mathrm{rec}}\in R^{B\times T\times F}$	Reconstructed input window	Section 3.2.1
${\hat{x}}^{\mathrm{fc}}\in R^{B\times F}$	Forecasted next time step	Section 3.2.1
β∈[0,1]	Weight between reconstruction and forecasting losses	Section 3.2.2
ρ=0.05	Trimming fraction applied to per-window losses	Section 3.2.2
$B_{\mathrm{rec}},B_{\mathrm{fc}}$	Trimmed sets of retained windows (rec./forecast.)	Section 3.2.2
*M*	Availability (alive) mask	Section 3.2.2 and Section 3.4.2
$\ell_{b}^{\mathrm{rec}},\ell_{b}^{\mathrm{fc}}$	Per-window reconstruction/forecasting loss	Section 3.2.2
$L_{\mathrm{rec}},L_{\mathrm{fc}}$	Trimmed batch losses	Section 3.2.2
L	Total multi-task training loss	Section 3.2.2
$s^{\mathrm{DL}}$	Combined (reconstruction + forecasting) anomaly score	Section 3.2.3
$s^{\mathrm{rec}},s^{\mathrm{fc}}$	Per-window reconstruction/forecasting scores	Section 3.2.3
$n_{f}$	Per-feature scaling factor of inference errors	Section 3.2.3
α=0.5	EMA decay rate applied to the reconstruction score	Section 3.2.3
τ	Detection threshold	Section 3.2.3
$y_{t}^{\mathrm{DL}}$	Binary DL anomaly label	Section 3.2.3
$cs_{t}^{\mathrm{DL}}$	Sigmoid-mapped confidence score of DL module	Section 3.2.3
$s_{\mathrm{tail}}$	Standard deviation of scores above τ	Section 3.2.3
*J*	Total number of expert-defined rules	Section 3.3
${\left\{r_{j}\right\}}_{j=1}^{J}$	Set of expert-defined rules	Section 3.3
$g_{j}\left(\cdot \right)$	Rule-specific activation function	Section 3.3
$z_{t,j}$	Scalar activation signal of rule *j* at time *t*	Section 3.3
$\theta_{j}$	Threshold/condition parameter of rule *j*	Section 3.3
$k_{j}$	Scaling factor (inverse std. dev. of the associated feature)	Section 3.3
$s_{t,j}$	Continuous confidence score of rule *j* at time *t*	Section 3.3
$s_{t}^{\mathrm{SEM}}$	Aggregated semantic anomaly score	Section 3.3
$y_{t}^{\mathrm{SEM}}$	Binary semantic anomaly label	Section 3.3
AP	Average Precision	Section 3.5
P@K, R@K	Precision/Recall at rank K	Section 3.5
*K*	Number of top-ranked windows evaluated (10% of test set)	Section 3.5
$N_{a}$	Number of injected synthetic anomalies	Section 3.5
AR	Rule activation rate	Section 3.5
Burst	Burstiness of rule activations	Section 3.5
NSI	Noise sensitivity index of a rule	Section 3.5
CI	Complementarity index between DL and semantic modules	Section 3.5
$\delta_{\mathrm{Cohen}}$	Cohen’s *d* effect size	Section 3.5

## Appendix B

This appendix focuses on the practical aspects of deploying the proposed AD framework in real-time monitoring environments at heritage sites.

To ensure reliable performance over time, scaler statistics and the active feature error set should be updated periodically, as sensor drift, calibration changes, and hardware replacements may alter the underlying data distribution. These updates should be computed exclusively from observations validated by the alive mask and not flagged as anomalous, preventing detected events from contaminating the reference distribution used for subsequent anomaly scoring. Significant changes in the active variable set may also require partial retraining of the spatial attention component to preserve the learning of inter-variable dependencies. The anomaly threshold τ is likewise sensitive to seasonal and long-term environmental variations. Periodic recalibration using Equation (7) on a recent set of non-flagged observations allows the threshold to adapt to evolving operating conditions, reducing the risk of excessive false alarms or missed detections. Finally, anomaly notifications should be managed at the event level rather than the individual window level. Consecutive anomalous windows associated with the same persistent phenomenon can be aggregated into a single alert using a minimum persistence criterion, consistent with the cooldown mechanism employed during anomaly injection. This strategy reduces alarm fatigue while preserving the ability to detect distinct events occurring at different times.

In practice, we recommend that the DL-side artifacts, namely the scaler statistics, the active feature set, and the detection threshold τ, be refreshed jointly on a monthly retraining cycle rather than updated independently, since these quantities are estimated from overlapping observation windows and can otherwise drift out of sync with one another, degrading the consistency of the anomaly score. Rule-specific thresholds $\theta_{j}$ follow a different logic and should only be revised when there is a concrete operational reason to do so. We propose recalibrating $\theta_{j}$ whenever: (i) the rolling activation rate of the corresponding rule (Section 3.5) deviates from its reference range over a sustained period (e.g., several consecutive days), indicating either sensor drift or a genuine change in the underlying environmental or structural regime; (ii) the associated sensor is physically replaced, relocated, or recalibrated, since this can shift both its absolute readings and its noise characteristics relative to the statistics used to compute $n_{f}$ and $\theta_{j}$; or (iii) a previously untested seasonal regime is observed for the first time at a site (Table 9), allowing rules that were regime-untestable during the original monitoring window to be validated once the relevant conditions occur. Conditions (i) and (ii) can be monitored automatically from existing pipeline outputs (rule-activation logs and sensor metadata), whereas (iii) requires a brief expert review triggered once per newly observed regime.

Regardless of the trigger, every recalibration event should be logged together with the previous and updated parameter values and a short justification (e.g., “scheduled monthly retrain”, “sensor S12 replaced on 4 March 2026”, “first freeze–thaw cycle observed at P5”). Maintaining this record is operationally important: because τ and $\theta_{j}$ directly determine what is reported as an alarm, undocumented changes to these parameters could make it difficult to distinguish whether a subsequent change in alarm rate reflects an actual change in site conditions or merely a change in system calibration.

## Appendix C

Table A2 summarizes the rules for the five pilot sites included in the semantic module.

**Table A2:** Summary of expert rules for all ARGUS datasets (P1–P5) divided by associated variables.

Variable	P1	P2	P3	P4	P5
Temp	-	-	change >5°C (1 h); ≥6 crossings of T=0°C (3 d) with RH >90%	$|T_{soil}-T_{air}|\;<1\;{}^{\circ}$C	≥6 crossings of T=0°C (3 d) with RH >90%
Humidity	>9 crossings around 75% (3 d)	daily range >10%	>97% ≥6 h; >90% ≥7 d; >20% (1 h); RH ≥90% with ≥6 crossings of T=0°C (3 d)	>80% inside	RH ≥90% with ≥6 crossings of T=0°C (3 d)
Wind	>12 m/s	-	>15 peak ratio	-	-
Rain	>10 mm/h; >20 mm (3 h)	-	>5 mm/h; >20 mm (24 h); >100 mm (7 d)	>50 mm (7 d)	-
Solar radiation	-	-	>3σ	-	-
Bar. pressure	-	-	range >900 Pa	-	-
Airflow	-	constant or daily range >6 m/s	-	-	-
Crackmeter	-	-	step change >90% (1 h); offset >0.6 (24 h)	-	step change >0.5 (1 h)
Tiltmeter	-	-	step change >90% (1 h); offset >60% (24 h)	-	-
Vibration	-	>0.25 PPV	>7σ	-	>5σ
GNSS	-	>0.03 mm daily change	-	-	-
Soil moisture	-	>67%; >2% daily change	-	>60% or <5%	-
People counter	-	-	≥30	-	-
Event dates	9 December 2025	13 February 2026	-	-	-

## Appendix D

Table A3 summarizes the training and evaluation configuration used for each baseline method. All deep learning models were trained for a maximum of 250 epochs with a patience of 20 epochs for early stopping. Hyperparameters were selected to be as similar as possible to those of NST-Net, ensuring comparability across methods. All models use the same preprocessed, globally standardized (1st/99th-percentile-clipped, without RevIN) feature set as NST-Net, restricted to the *F* sensor variables. The six temporal context features ($F_{c}=6$) and the alive mask are not provided to the baselines, as their original implementations do not support these inputs. Missing observations were replaced with a constant value for all baselines, since their original implementations do not support masked optimization or availability-aware normalization. To further assess the effect of this preprocessing, we include NST-Net-bp, a variant of NST-Net using the same baseline preprocessing.

**Table A3:** Training and evaluation configuration per baseline method. *B*: batch; *T*: lookback window length; *H*: hidden/latent dimension.

Method	*B*	*T*	Epochs/Patience	Key Hyperparameters
IForest	–	72	–	window flattened to T×F input; ρ=0.05
USAD	256	72	250/20	*H* = 32; $w_{\mathrm{size}}=T\cdot F$, $z_{\mathrm{size}}=T\cdot H$
MTAD-GAT	256	72	250/20	*H* = 32; Adam, lr $=10^{-3}$, wd $=10^{-4}$
GDN	256	72	250/20	*H* = 32; static learned graph k=5
AnomTr	256	72	250/20	k=3; anormly_ratio =1.0; lr $=10^{-4}$
TimesNet	256	72	250/20	*H* = 32

## Appendix E

Figure A1 shows six types of synthetic anomalies injected across different features.

The collective shift causes a sustained level change, while the variance burst temporarily increases variability. The frozen anomaly reduces dynamics within the affected window, whereas the contextual anomaly is abnormal relative to its temporal position (24 h periodicity). Spikes and drops represent isolated positive and negative point anomalies, respectively. The drop example also illustrates that unlabeled data points are not necessarily normal. Algorithm A1 summarizes the injection pipeline, with anomalies grouped into collective, contextual, and point types. Figure A1 shows the original series and injected anomalies.

**Algorithm A1** Synthetic anomaly injection	
**Require:** Test values $X\in R^{N\times F}$; train+val values $X_{tv}$ and alive mask $M_{tv}$; alive mask *M*; period $T_{p}$; frequency freq; severity sev; correlation threshold γ; max partners $p_{\max}$; cooldown parameter *C*	
**Ensure:** Perturbed series $\tilde{X}$, window-level labels *y*	
1:	$\tilde{X}\leftarrow X$; O← False ▹occupancy mask
2:	$n_{f}\leftarrow Q99\left(X_{tv}\left[M_{tv}\;=\;1\right]\right)-Q1\left(X_{tv}\left[M_{tv}\;=\;1\right]\right)$ for each feature *f* ▹ global per-feature scale, train+val only
3:	R← pairwise absolute correlation matrix of *X* over non-constant features
4:	P← features with strong lag-$T_{p}$ autocorrelation; for each f∈P, locate local peaks/troughs (donor candidates)
5:	k←⌊freq·N/3⌋; set target counts $\left\{k_{\mathrm{coll}},k_{\mathrm{ctx}},k_{\mathrm{pt}}\right\}$ per type
6:	$L_{c}\leftarrow 12$ (collective length), $L_{x}\leftarrow 3$ (contextual length)
7:	**function** Partners(*f*)
8:	**return** up to $p_{\max}$ features *g* with $R_{f,g}\geq \gamma$, ranked by correlation
9:	**end function**
10:	**function** TryCollective
11:	Sample start *s*, feature *f*; let $e\leftarrow s+L_{c}$
12:	**if** region [s,e] free and *f* alive **then**
13:	Cycle mode ∈{shift,varianceburst,frozen} (round-robin, no repeats until all used)
14:	Perturb $\tilde{X}\left[s:e,f\right]$ by magnitude $sev\cdot n_{f}$ per mode; repeat on each feature in Partners(*f*) using its own $n_{g}$
15:	y[s:e]←1; mark [s−C,e+C] occupied; **return** success
16:	**end if**
17:	**end function**
18:	**function** TryContextual
19:	Draw next unused (feature f∈P, extremum *c*) pair; let [s,e]←[c−1,c+2]
20:	**if** region free and a same-feature donor extremum *d* of *opposite* phase exists with $\left|d-c\right|\geq 2L_{x}$ and correlation$(\tilde{X}\left[s:e,f\right],X\left[d\;-\;1:d\;+\;2,f\right])\leq 0.85$ **then**
21:	Replace $\tilde{X}\left[s:e,f\right]$ with donor window X[d−1:d+2,f]; repeat for each partner in Partners(*f*) using its matching-phase donor
22:	y[s:e]←1; mark [s−C,e+C] occupied; **return** success
23:	**end if**
24:	**end function**
25:	**function** TryPoint
26:	Sample timestamp *t*, feature *f*
27:	**if** M[t,f]=1 and *t* free **then**
28:	Cycle mode ∈{spike,drop} (round-robin); set $\tilde{X}\left[t,f\right]\leftarrow \tilde{X}\left[t\;-\;1,f\right]\pm sev\cdot n_{f}$; repeat on each partner in Partners(*f*)
29:	y[t]←1; mark [t−C,t+C] occupied; **return** success
30:	**end if**
31:	**end function**
32:	**Round-robin scheduling:** while any active type is below its target count and below a failure-streak budget, cycle through {collective, contextual, point} in turn, attempting one placement per active type per pass; deactivate a type once its target count is reached or its failure streak is exceeded
33:	Assign each raw anomalous timestep as the positive label of its corresponding window; under this convention, window-level and point-level labels coincide one-to-one
34:	**return** $\tilde{X},y$

## Appendix F

This appendix focuses on the initial evaluation of NST-Net performance on slow and cumulative anomalies. To this end, we injected one anomaly at each site, with a duration corresponding to 40% of the test set length. The injection pipeline increases the magnitude of the deviation linearly over the anomaly duration, starting from the original data point at the onset of the anomaly and reaching a value of $sev\cdot n_{f}$ at its end. Table A4 reports the AP results, compared against the default 5% mixed-anomaly injection used throughout this paper, alongside the anomaly duration. The duration values explain why this type of anomaly was not adopted in our original evaluation pipeline. Even when the injected slow drift is allowed to occupy 40% of the test period, its duration remains below 12 days at all sites and less than 2 days for P5. In addition, having almost half of the test set as anomalies is quite uncommon due to the nature of anomalies themselves and hinders comparison with lower anomaly ratios. Because AP is sensitive to positive-class prevalence (Section 4.3), the raw AP values at 40% cannot be compared directly to those at 5%.

**Table A4:** Realized injected ratios and performance in terms of AP for different targeted anomaly ratios across the five pilot sites. For the 40% target, the duration of injected anomaly points is additionally reported in hours and days. Arrows indicate the desired values.

	P1		P2		P3		P4		P5	
Targeted	Injected	AP↑	Injected	AP↑	Injected	AP↑	Injected	AP↑	Injected	AP↑
5.00%	4.43%	0.322 ±0.073	4.07%	0.082 ±0.031	4.36%	0.176 ±0.087	4.16%	0.119 ±0.049	11.82%	0.217 ±0.107
40.00%	40.00% 248 h/10.33 d	0.393 ±0.090	40.00% 131.5 h/5.48 d	0.401 ±0.066	40.00% 262.5 h/10.94 d	0.430 ±0.036	40.00% 280.5 h/11.69 d	0.409 ±0.040	40.00% 38.5 h/1.60 d	0.529 ±0.002

To assess model performance under this anomaly type while eliminating the effect of the realized injection ratios, we also report in Table A5 a prevalence-normalized AP:

(A1) $$\mathrm{nAP}=\frac{\mathrm{AP}-p}{1-p},$$

where *p* denotes the realized anomaly prevalence. This measure accounts for the prevalence-dependent random-ranking baseline, for which AP≈p, and therefore facilitates a more meaningful comparison across different anomaly ratios. In particular, nAP=0 corresponds to random-ranking performance, while nAP=1 corresponds to perfect ranking. While raw AP appears higher under the 40% slow-drift injection than under the default 5% protocol at every site, the prevalence-normalized nAP tells a different story: nAP drops sharply and is close to zero or negative at four of the five sites (P1–P4), indicating that once the much higher positive-class prevalence is accounted for, NST-Net’s ranking of slow-drift windows is barely better than random and markedly worse than its ranking of the short, sharp anomalies evaluated in Section 4.1, Section 4.2 and Section 4.3. P5 is the exception, though given its short test period and the resulting small number of anomalous windows, this estimate should be interpreted with the same caution already noted.

**Table A5:** Comparison of AP and prevalence-normalized AP (nAP) between the 5% and 40% anomaly injection levels across the five pilot sites. The normalized AP is defined as nAP=(AP−p)/(1−p), where *p* denotes the realized anomaly ratio.

	5%		40%		Change	
Site	AP	nAP	AP	nAP	ΔAP	ΔnAP
P1	0.322	0.286	0.393	−0.012	+0.071	−0.299
P2	0.082	0.034	0.401	0.000	+0.319	−0.033
P3	0.176	0.133	0.430	0.048	+0.254	−0.084
P4	0.119	0.073	0.409	0.015	+0.290	−0.058
P5	0.217	0.112	0.529	0.213	+0.312	+0.101

These results provide initial, quantitative support for the qualitative limitation noted in Section 5: NST-Net’s detection advantage is concentrated on sharp, short-duration deviations, and its sensitivity to slow, cumulative drift is substantially weaker.

## Appendix G

Table A6 reports the median activation rate (AR), together with the native interquartile range, obtained when each site’s expert-rule set is applied unmodified to the data from every other site. The results show that activation rates can shift substantially relative to their native calibration and in either direction. Most transferred rules become essentially inactive, with median ARs and interquartile ranges close to zero, whereas a smaller subset becomes markedly more permissive than at its site of origin. For example, when rules from P1 are transferred to P2 or P4, the median AR significantly exceeds 0.1, with Q3 values approaching 1. In contrast, most of the remaining transfers yield median ARs close to zero, often accompanied by very narrow interquartile ranges. Nevertheless, some transfers exhibit a more heterogeneous response: in particular, rules transferred between P2 and P4, as well as from P2 to P5, show relatively high Q3 values, indicating that although the overall behavior is restrictive, a subset of rules can become excessively permissive under transfer.

Importantly, a substantial fraction of the near-zero activation rates should not necessarily be interpreted as evidence that the transferred rules become more restrictive. In many cases, they reflect structural inapplicability, whereby the variable underlying a rule is simply unavailable at the target site (rules applied to sites without the corresponding sensors). Table A2 in Appendix C provides an overview of the variables available at each site and can therefore be used to distinguish such cases of structural inapplicability from genuine changes in rule behavior under transfer.

**Table A6:** Pairwise median AR values between sites, with interquartile ranges (Q1–Q3). Columns indicate the origin site (From →), and rows indicate the target site (To ↓). In bold are the native median and interquartile ranges (Q1–Q3) for each site.

From →	P1	P2	P3	P4	P5
To ↓					
**P1**	**0.031 [0.011–0.034]**	0.000 [0.000–0.000]	0.002 [0.000–0.013]	0.000 [0.000–0.001]	0.000 [0.000–0.002]
**P2**	0.403 [0.001–0.950]	**0.001 [0.000–0.022]**	0.000 [0.000–0.000]	0.000 [0.000–0.189]	0.000 [0.000–0.000]
**P3**	0.000 [0.000–0.001]	0.000 [0.000–0.001]	**0.054 [0.004–0.070]**	0.000 [0.000–0.009]	0.000 [0.000–0.002]
**P4**	0.712 [0.127–0.973]	0.000 [0.000–1.000]	0.000 [0.000–0.000]	**0.005 [0.000–0.060]**	0.000 [0.000–0.000]
**P5**	0.000 [0.000–0.008]	0.000 [0.000–0.233]	0.000 [0.000–0.068]	0.000 [0.000–0.071]	**0.023 [0.004–0.029]**

This study confirms that even when a rule is structurally transferable, its expert-calibrated threshold does not generalize across sites without site-specific recalibration, consistent with the site specificity discussed in Section 5.
